# Specific interactions between fluorinated vitamin-D_3_ derivatives and vitamin-D receptor: molecular mechanics and ab initio fragment molecular orbital calculations

**DOI:** 10.1007/s00894-025-06623-1

**Published:** 2026-01-14

**Authors:** Masayuki Yuguchi, Shuta Takenaka, Chisato Nakatani, Yoshinobu Nagura, Haruna Sabishiro, Nagomi Chimura, Atsushi Kittaka, Midori Takimoto-Kamimura, Noriyuki Kurita

**Affiliations:** 1 Department of Computer Science and Engineering, Toyohashi University of Technology, Tempaku-Cho, Toyohashi, Aichi 441-8580 Japan; 2 Faculty of Pharmaceutical Sciences, Teikyo University, 2-11-1 Kaga, Itabashi, Tokyo 173-8605 Japan; 3 Quantum-Structural Life Science Laboratories, CBI Research Institute, Kyowa Create Daiichi Build. 3 F, 3-11-1 Shibaura Minato, Tokyo, 108-2234 Japan

**Keywords:** Vitamin D receptor, Inhibitor, Fluorinated compound, Diastereomers, Molecular simulation, Fragment molecular orbital, Protein–ligand interaction, Binding affinity

## Abstract

**Context:**

Various vitamin D_3_ (VD3) derivatives have been developed as potent inhibitors against vitamin D receptor (VDR) for blocking the specific binding of active vitamin D to VDR. Among them, some fluorinated VD3 derivatives were revealed to possess an improved binding affinity to VDR. However, the reason for this significant improvement has not been elucidated. In the present study, we investigated the specific interactions between VDR and the fluorinated VD3 derivatives using molecular mechanics and ab initio fragment molecular orbital (FMO) calculations. Additionally, we considered the diastereomers based on both C3- and C13-stereocenters of these VD3 derivatives and investigated their interactions with VDR, elucidating that the evaluated binding energies between VDR and the diastereomers are comparable to the trend of their binding affinities to VDR obtained by the previous experiment. Based on the FMO results, the effect of fluorination of VD3 derivatives on their specific interactions with VDR was also elucidated at atomic and electronic levels. The present finding may offer valuable insights for proposing novel inhibitors targeting VDR.

**Methods:**

The structures of our target VD3 derivatives were optimized using the B3LYP/6-31G(d,p) method of Gaussian 16 (G16). The charge distributions of the optimized structures were calculated by constrained electrostatic potential (RESP) analysis using the HF/6-31G(d) method of G16. The RESP charges were used for describing the electrostatic interactions between each derivative and the VDR residues in classical molecular mechanics (MM) and molecular dynamics (MD) calculations. As the initial structure of VDR, the X-ray crystal structure (PDB ID: 1DB1) was used, and by using the fitting tool (gmx confrms) of the MD simulation program GROMACS, the initial structures of the VDR − derivative complexes were created. To obtain their stable structures, the classical MM method of AMBER18 was used. The tleap command of AMBER18 was employed for adding hydrogen and fluorine atoms to the PDB structure and generating solvation water molecules within 8 Å around the complex. This solvated structure of the complex was optimized using the MM method by considering water molecules explicitly. In the MM optimizations, the AMBER14SB force field, the generalized AMBER force field, and the TIP3P model were assigned for VDR, derivatives, and water molecules, respectively. We furthermore conducted MD simulations (100 ns at 300 K) for the MM optimized structures using the same force fields and confirmed the stability of the complex. Finally, to elucidate the specific interactions between VDR and each VD3 derivative at an electronic level, we investigated the electronic properties of the complexes in explicit water using the ab initio fragment molecular orbital (FMO) method. We employed the ab initio MP2/6-31G(d) method of the FMO calculation program ABINIT-MP Ver6.0, to accurately investigate the π–π stacking, NH–π, and CH–π interactions as well as the hydrogen-bonding and electrostatic interactions between the VDR residues and the derivatives. Furthermore, to highlight the critical VDR residues for the binding between VDR and the derivative, we investigated the inter-fragment interaction energies obtained by the FMO calculations.

**Supplementary Information:**

The online version contains supplementary material available at 10.1007/s00894-025-06623-1.

## Introduction

**Fig. 1:**
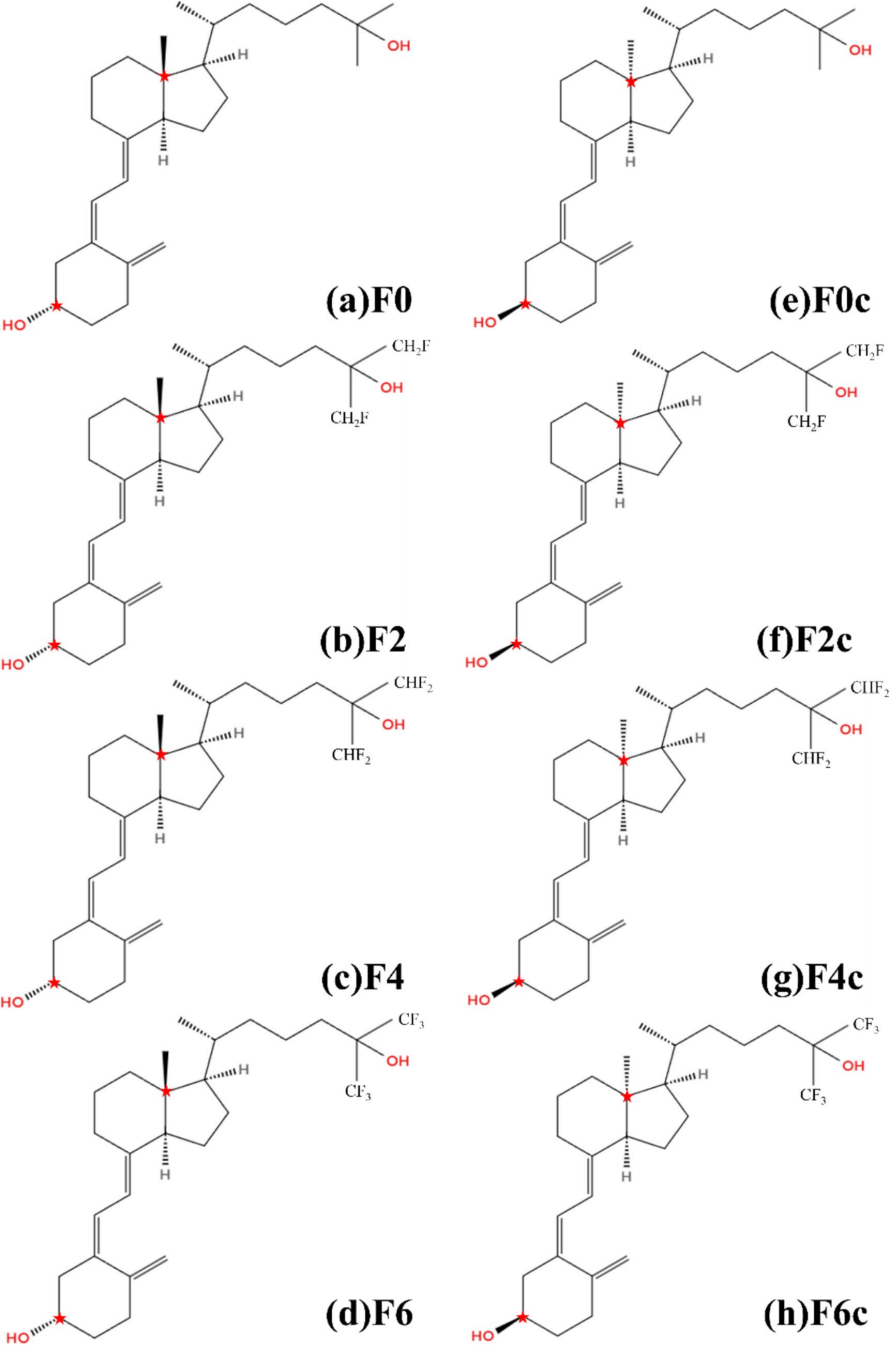
Chemical structures of the active vitamin D3 derivatives employed in the present study. Based on (**a**) **F0** structure, (**b**) **F2**, (**c**) **F4**, and (**d**) **F6** are fluorinated at the C-26 and C-27 sites. (**e**) **F0c**, (**f**) **F2c**, (**g**) **F4c**, and (**h**) **F6c** are their diastereomeric forms, in which the configurations at both C-3 and C-13 sites indicated by red stars are different

Vitamins act like lubricants, facilitating the metabolism of energy-producing nutrients in the body: carbohydrates, fats, and proteins. Among them, vitamin D (VD) is a generic term that includes vitamin D_2_ (ergocalciferol) and vitamin D_3_ (cholecalciferol); vitamin D_2_ is produced from ergosterol found in plants, and vitamin D_3_ is produced by exposing 7-dehydrocholesterol found in animals to UV light. Vitamin D_3_ is hydroxylated in the liver and kidneys at the 25 and 1α sites to form the active form of vitamin D_3_, 1α,25-dihydroxyvitamin D_3_ (1α,25(OH)_2_D_3_). In this paper, we employed 25-hydroxyvitamin D_3_ (25(OH)D_3_) and denoted it as **F0**, and its structural formula is shown in Fig. 1a. **F0** has been found to play an essential role in the metabolism of calcium and phosphorus and regulation of bone formation [1–5]. Deficiency of **F0** can lead to abnormal bone formation, which may cause rickets in children and increase the risk of osteomalacia in adults [6].

The vitamin D receptor (VDR) is a type of nuclear receptor that forms dimers via specific binding to **F0** and regulates the expression of target genes by specifically binding to the vitamin D response sequence in the gene [7]. The target genes also include genes that induce cell differentiation and cell death. VDRs are present in almost all cellular tissues in vivo, and the association between various pathological conditions and genetic variation in VDRs has been widely studied [8]. VDRs mediate the pleiotropic biological actions of 1α,25(OH)_2_D_3_, which include orchestration of mineral homeostasis coordinated by the kidney, intestine, bone, and parathyroid gland.

Inhibitors against VDR are expected to be effective therapeutic agents for bone Paget’s disease, which occurs when the sensitivity of VDR to **F0** is abnormally increased, and for hypercalcemia, which is caused by vitamin D overload and other factors. Previous experiments [9] have shown that introducing fluorine atoms into **F0** significantly increases its binding affinity to VDR. However, the cause of this increase is still unknown and remains a bottleneck in developing more effective inhibitors against VDR.

Introducing a fluorine atom, with the highest electronegativity among all types of atoms, into **F0** can induce many electrons around it, thereby changing the charge distribution within **F0** and significantly changing the electrostatic interaction between **F0** and VDR. Furthermore, as the binding energy of the C − F bond (485 kJ/mol) is higher than that of the C − H bond (414 kJ/mol), more stable inhibitors can be realized by introducing a fluorine atom into **F0**. In the previous studies [9–12], compounds with fluorine atoms introduced at the positions 1 to 11 and 22 to 27 of **F0** were synthesized, and their binding properties to VDR were investigated. As a result, it was found that compounds with fluorine atoms introduced at the positions 26 and 27 of **F0** bind strongly to VDR [9]. In fact, the compound **F6** (Fig. 1d), in which methyl groups at the positions 26 and 27 were substituted by trifluoromethyl units, showed a 57-fold increase in binding affinity to VDR compared to **F0**. On the other hand, the compound **F2** (Fig. 1b), in which one hydrogen atom of the methyl groups at the positions 26 and 27 were substituted by a fluorine atom, showed almost the same binding affinity as **F0**. The compound **F4** (Fig. 1c), in which two hydrogen atoms of the methyl groups were substituted by fluorine atoms, exhibited a higher binding affinity than **F0** but lower binding affinity to VDR than **F6**. Thus, the binding affinity of these fluorinated compounds to VDR was found to vary greatly depending on the number of fluorine atoms introduced at the positions 26 and 27 of **F0**. Therefore, it is expected to be possible to propose novel inhibitors that can bind more strongly to VDR by optimizing the number and position of fluorine atoms to be introduced into **F0**. However, the reason for the change in binding properties to VDR due to fluorine substitutions remains unclear.

In the present study, to elucidate the cause of the above experimental results, we investigated specific interactions between VDR and **F0** and its fluorinated compounds at the electronic level using ab initio molecular simulations based on the fragment molecular orbital (FMO) method. As target inhibitors, we employed **F0** and its fluorinated compounds **F2**, **F4**, and **F6**, whose chemical structures are shown in Figs. 1a − 1d. Notably, we additionally considered their diastereomeric forms (**F0c**, **F2c**, **F4c**, and **F6c**). As shown in Figs. 1e − 1 h, these diastereomeric compounds have different configurations at both C-3 and C-13 sites in comparison with their original forms. Based on the ab initio FMO calculations, how the fluorine substitution changes the electrostatic interaction between **F0** and VDR and improves the binding affinity to VDR was elucidated at the electronic level. Furthermore, we investigated how the configurational change of the fluorinated compounds affects their specific interactions with VDR.

### *Details of molecular simulations*

#### Construction of structures of VDR − compound complexes

Gauss View software [13] was used to make the initial structures of our target inhibitors shown in Fig. 1. The structures were optimized in vacuum using the B3LYP/6-31G(d, p) method of the ab initio molecular orbital calculation program Gaussian 16 (G16) [14]. The charge distributions of the optimized structures were calculated by constrained electrostatic potential (RESP) analysis [15] using the HF/6-31G(d) method in G16. These methods were used to obtain charge parameters of inhibitors in a consistent manner as the AMBER force fields of the amino acid residues of VDR. RESP charges are essential for accurately describing the electrostatic interactions between each compound and the amino acid residues of VDR in classical molecular mechanics (MM) and molecular dynamics (MD) calculations.

In the present study, the X-ray crystal structure registered in the Protein Data Bank (PDB) (PDB ID: 1DB1 [16]) was used as the initial structure of VDR. Although VDR consists of 259 amino acid residues, this PDB structure lacks positional information for the amino acid residues from 374 to 377th of VDR. To compensate for these missing residues, we compared some PDB structures of VDR, using the fitting tool (gmx confrms) included in the MD simulation program GROMACS [17]. As the sequences of amino acids in the 1DB1 and 3P8X PDB structures are similar, the 3P8X structure was employed for fitting. Only the main chain of the VDR structures were fitted, and the PDB structure of 3P8X was copied to the missing part of the 1DB1 structure to create the initial structure of VDR without the defect.

The initial position of the compound in the VDR − compound complex was set to be the same as the position of the compound in the PDB structure of 1DB1, because the chemical structures of our target compounds (**F0** and **F0c**) shown in Fig. 1 are almost the same as the compound in the PDB structure. The initial structure of each fluorinated compound was created by substituting hydrogen atoms with fluorine atoms based on the structure of **F0** or **F0c**. Notably, we here employed the initial structures of the VDR − compound complexes, which were created by fitting to the experimentally obtained PDB structure, because our target compounds have similar structures as the compound in the PDB structure. In our previous study [18], the same procedure based on fitting was employed for constructing initial structures of the VDR − compound complexes, and the binding energies evaluated using FMO calculations between VDR and the compounds were confirmed to be comparable to the trend of binding affinities obtained by experiments. Accordingly, we used the same fitting method in the present study. To verify the validity of the fitting method, we also docked some compounds to VDR using a protein–ligand docking program and compared the docked structures with the structures created through fitting.

To obtain the stable structure of the complex of VDR and each compound, the classical MM method of AMBER18 [19] was used. For system preparation prior to the MM simulations, the tleap command of AMBER18 [19] was employed for adding hydrogen and fluorine atoms to the PDB structure and generating solvation water molecules within 8 Å around the complex. This solvated structure of the complex was optimized using the MM method by considering water molecules explicitly. In the MM optimizations, the AMBER14SB force field [20], the generalized AMBER force field (GAFF) [21], and the TIP3P model [22] were assigned for VDR, compounds, and water molecules, respectively. The convergence criterion for structural optimization was set as 0.0001 kcal/mol/Å.

To confirm the stability of the structure of complex optimized using the MM method, we carried out 100 ns MD simulations in explicit water and investigated the structural change of the complex. The MD simulations were carried out in a cuboidal water box, whose size was determined as the distance between the surface of the complex and the boundary of the box is 10 Å, and the complex was initially placed at the center of the box. The MD simulations using GROMACS [17] were executed by setting the periodic boundary condition in the XYZ direction with the water box as a unit cell. We first optimized the solvated structure of the complex by the energy minimization method of GROMACS. Subsequently, structural equilibrium calculations were executed by 1 ns MD simulations under constant temperature and pressure conditions (300 K, 1 bar), to relax the position and the density of the solvating water molecules. After optimizing the size of the water box, 100 ns MD simulations were conducted under constant temperature (300 K) and volume condition.

#### Analysis of specific interactions between VDR and compounds using FMO

To elucidate the specific interactions between VDR and each compound, we investigated the electronic properties of the complexes in explicit water using the ab initio FMO method [23–25], in which the target large molecule is divided into fragments and its electronic properties are estimated from the electronic properties of the monomers and the dimers of the fragments. In the present FMO calculations, we considered explicitly the water molecules existing within 8 Å from the compound of the complex to properly describe the effect of water molecules on the specific interactions between VDR and each compound. We employed the ab initio MP2/6-31G(d) method of the FMO calculation program ABINIT-MP Ver6.0 [26], to accurately investigate the π–π stacking, NH–π, and CH–π interactions as well as the hydrogen-bonding and electrostatic interactions between the VDR residues and the compound. We here employed the small 6-31G(d) basis-set, as MP2 calculations with larger basis-set are not practical for the VDR–compound complexes. To confirm the basis-set dependence of the interactions between VDR and compound, we also conducted MP2 calculations using the 6-31G and 6-31G(d,p) basis-sets. Each fragment was assigned to each amino acid residue of VDR, the compound, and each water molecule, because this fragmentation makes it possible to analyze the interactions between the VDR residues and the compound. Furthermore, to highlight the critical VDR residues for the binding between VDR and the compound, we investigated the inter-fragment interaction energies (IFIEs) [27] obtained by the FMO calculations.

In the present study, we did not consider the effect of entropy on the binding affinity because the vibrational analysis for the solvated VDR − compound complex is not practical by the ab initio FMO method and because the entropic effect is likely to be not so different for each of the fluorinated VD3 derivatives, which have almost the same chemical structures as shown in Fig. 1. Therefore, we investigated binding energies between VDR and the derivatives using the ab initio FMO calculations, estimating the trend of binding affinity under the assumption that the entropic effect remains the same for the derivatives.

## Results and discussion

### Determination of His protonation states of VDR using FMO calculations

The X-ray crystal structure of VDR (PDB ID: 1DB1 [16]) does not include the positions of hydrogen atoms. Therefore, it is necessary to add hydrogen atoms to the appropriate positions for conducting molecular simulations. Notably, some of the 20 amino acids that comprise a protein can bond with hydrogen atoms in multiple ways. Specifically,　histidine (His) residues can exhibit three protonation states, as shown in Figure S1 of Supplementary Information (SI). However, the specific state depends on the pKa value of each His residue and its position relative to the surrounding amino acids. In fact, VDR contains nine His residues, and it is difficult to determine the protonation states of these His residues by experiment alone. In the present study, we assumed several possible protonation states to create several initial structures of the complex and evaluated the total energies (TEs) of these optimized structures using FMO calculations to determine the most stable protonation state. The protonation states of the seven His residues in VDR could be uniquely determined by the structures of the surrounding His residues as indicated in Table S1 of SI. The remaining two His residues (His305 and His397) existing inside VDR can either be in the Hid or Hie protonated state. Therefore, four initial structures were created considering the combination of the four His protonation states of His305 and His397. These structures were then optimized using the MM calculations, and the TEs were calculated using ab initio FMO calculations.

Table S2 of SI compares the evaluated TEs, indicating that the change in the range of TE for the VDR − **F0** and VDR − **F6** complexes is less than 0.1 kcal/mol, even if the protonation states of His305 and His397 change between Hid and Hie. It was also found that the Hid/Hid protonation state combination is the most stable protonation state for the VDR complexes containing **F0** or **F6**. Therefore, the subsequent molecular simulations were performed with the protonation states of His305 and His397, both set to Hid. Notably, these His residues exist near the compound in the VDR − compound complexes to contribute significantly to the interactions between VDR and compound.

### Binding properties between VDR and VD3 derivatives

**Fig. 2:**
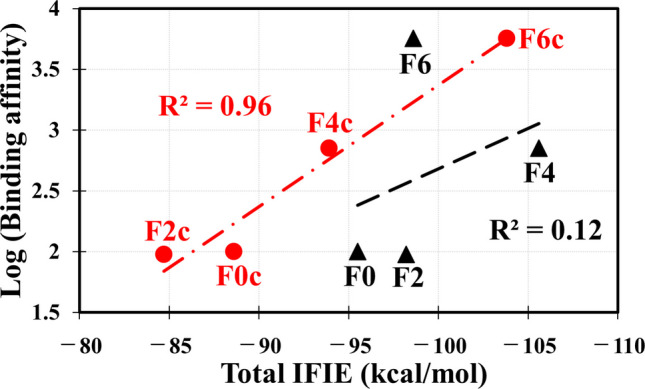
Correlation between the binding affinities of the fluorinated compounds shown in Fig. 1 obtained in the previous experiment [9] and the total IFIEs evaluated using the present FMO method between each compound and all VDR residues. A black line is for the **F0**, **F2**, **F4**, and **F6** compounds; a red line is for the diastereomeric compounds (**F0c**, **F2c**, **F4c**, and **F6c**)

First, we investigated binding properties between VDR and **F0** (Fig. 1a) in the PDB structure (PBD ID: 1DB1 [16]) and the fluorine substituted derivatives (**F2**, **F4**, and **F6**: Figs. 1b, 1c, and 1 d) based on **F0**, using ab initio FMO calculations. Table 1 compares the evaluated total IFIEs between each derivative and all amino acid residues of VDR and the relative binding affinities obtained in the previous experiment [9]. The results of the correlation analysis between the evaluated and experimental values for the **F0**, **F2**, **F4**, and **F6** compounds having the same configuration are marked by a black line in Fig. 2; the correlation coefficient between them is R^2^ = 0.12. The previous experiment [9] reported that the binding affinity between VDR and **F0** was increased 57-fold by introducing six fluorine atoms. On the other hand, the present FMO calculations for the four compounds with the configuration shown in Figs. 1a–1d revealed that **F4** binds more strongly to VDR compared with **F6**. Therefore, the size of the total IFIEs evaluated for the compounds (**F0**, **F2**, **F4**, and **F6**) could not explain the trend of the binding affinity of each compound to VDR.

**Table 1:** Relative binding affinities between VDR and the compounds (**F0**, **F2**, **F4**, and **F6)** obtained by the previous experiment [9], and total IFIEs (kcal/mol) evaluated using the present FMO method between all VDR residues and each of the compounds. Total IFIEs for their diastereomeric forms (**F0c**, **F2c**, **F4c**, and **F6c**) are also listed for comparison. Their chemical structures are shown in Fig. 1

Compound	**F0**	**F2**	**F4**	**F6**
Binding affinity (%)	100	95	710	5700
Total IFIE	− 95.5	− 98.2	− 105.6	− 98.6
Compound	**F0c**	**F2c**	**F4c**	**F6c**
Total IFIE	− 88.6	− 84.7	− 93.9	− 103.8

As a possible reason why the results of the FMO calculations could not explain the trend of the experimental results, we considered the possibility that the compound may bind to the ligand-binding pocket of VDR with a structure of different configuration from the structure shown in Figs. 1a–1d, because our previous study [18] for VDR complexes with other types of VD3 derivatives confirmed that the trend of evaluated total IFIEs between VDR and the derivatives can explain the trend of binding affinities obtained by experiments. Additionally, the entropic effect is likely to be not so different for each of the fluorinated derivatives, which have almost the same chemical structures as shown in Fig. 1. Therefore, we created a different structure for each of the VD3 derivatives, by changing the configurations at the C-3 and C-13 sites as shown in Figs. 1e–1h. The C-3 site was selected because it has a hydroxy group and the change in C-3 configuration is expected to influence the interactions between the derivatives and VDR. Additionally, we selected the C-13 site and changed the configuration as it has an opposite configuration from the C-3 site in the original compounds and the total numbers of R- and S-configurations in each compound are not changed by the configuration chang at both C-3 and C-13 sites. The binding properties between these diastereomeric compounds (**F0c**, **F2c**, **F4c**, and **F6c**) and VDR were investigated using the same molecular simulations as used for the VDR–**F0** complex.

The structures of these compounds were optimized using the B3LYP/6-31G(d, p) method of G16 [14], and their TEs were compared to those for the original compounds. As indicated in Table S3 of SI, the TE for each compound became about 8 ~ 15 kcal/mol higher due to the change in configurations at the C-3 and C-13 sites. This change in TE is considered to be caused mainly by a change in the relative positions of the two OH groups at both ends of the compounds. When each compound exists alone in vacuum, this significant difference in TEs suggests that structures with different configurations are unlikely to exist stably. On the other hand, there is a chance that the compounds can have structures with a different configuration from **F0** in the binding pocket of VDR under the effect of VDR residues existing around the pocket. Therefore, we investigated the interactions between VDR and the diastereomeric compounds (**F0c**, **F2c**, **F4c**, and **F6c**) shown in Figs. 1e–1h. From the comparison between the experimental and evaluated results, we attempted to determine which configuration of the compounds could be more realistic in their complexes with VDR.

The four compounds (**F0c**, **F2c**, **F4c**, and **F6c**) shown in Figs. 1e–1h were fitted to the binding pocket of VDR in the same manner as the VDR–**F0** complex, and the structures of the VDR–compound complexes were optimized in water using the classical MM method to investigate the binding properties between VDR and each compound using FMO calculations. To verify the validity of the fitting method, we also docked **F0c** and **F6c** to the VDR ligand-binding pocket using the automated protein–ligand docking program AutoDock [28] and compared the docked structures with that created through the fitting method. The root-mean-square-deviations (RMSD) between the structures of each ligand are 1.6 Å (**F0c**) and 2.0 Å (**F6c**), respectively. We also compared the conformations of the ligands, confirming that the binding conformations of the ligands to VDR are similar in the fitting and docking simulations.

The evaluated total IFIEs were compared to the experimental results [9] in Table 1 and Fig. 2. We selected **F0c**, **F2c**, **F4c**, and **F6c** compounds having the same configuration and obtained the correlation coefficient (R^2^ = 0.96) between these results, as indicated by a red line in Fig. 2. By adopting the structures of the compounds with different configuration shown in Figs. 1e–1h, we could reproduce the trend of binding affinities of the compounds to VDR obtained in the experiment [9]. Accordingly, we would suggest the probability that the fluorine substituted compounds can have a different configuration in the binding pocket of VDR.

To examine the basis-set dependence of the interactions between VDR and compound, we also conducted FMO MP2 calculations using the 6-31G and 6-31G(d,p) basis-sets and analyzed the total IFIEs between VDR and **F0c** or **F6c**. The results elucidated that the difference in total IFIEs of **F0c** and **F6c** is 11.2 (6-31G), 15.2 (6-31G(d)), and 15.3 (6-31G(d,p)) kcal/mol, respectively, indicating that this value is almost the same in the MP2 calculations using the 6-31G(d) and 6-31G(d,p) basis-sets. Therefore, it can be considered that the relative binding energy between VDR and **F0c/F6c** can be estimated using MP2/6-31G(d) calculations. In the present study, we employed the 6-31G(d) basis-set in the FMO MP2 calculations.

**Fig. 3:**
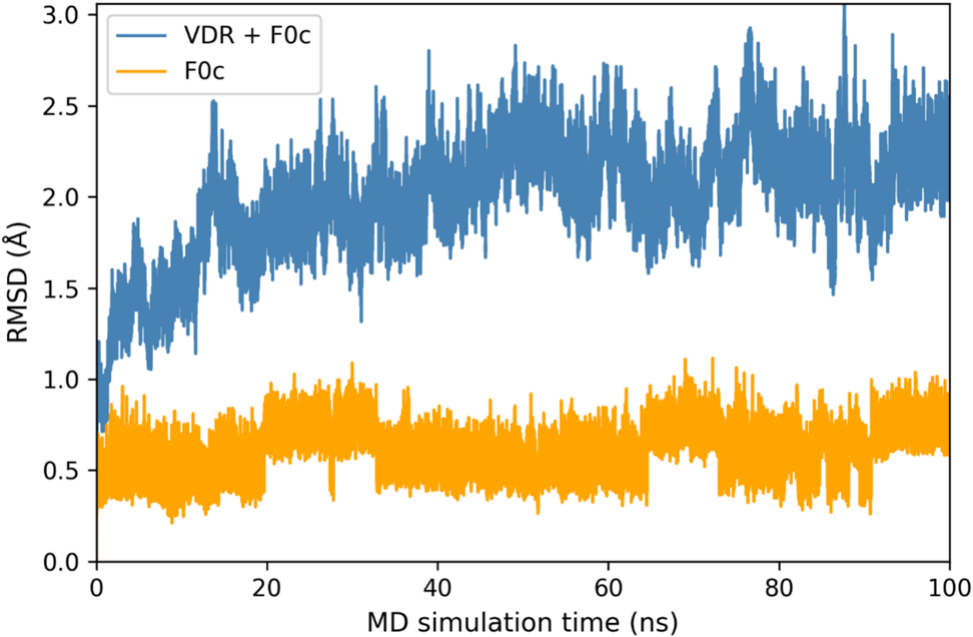
Root mean square deviation (RMSD) between the MM-optimized and MD snapshot structures of VDR − **F0c** complex during 100 ns MD simulations; orange line indicates RMSD of all heavy atoms of **F0c**, and blue line indicates RMSD of all backbone atoms of VDR and **F0c**

To confirm the stability of the complexes, we moreover conducted preliminary MD simulations (100 ns at 300 K) for the MM optimized structure of the VDR–**F0c** complex. The RMSD between the MM-optimized and MD snapshot structures is shown in Fig. 3, indicating that the size of RMSD for **F0c** is smaller than 1.0 Å and nearly constant during the MD simulations, and the RMSD for the VDR–**F0c** complex is smaller than 3.0 Å and does not change significantly. Therefore, the structure of the VDR–**F0c** complex was confirmed to be stable. The results of MD simulations for the other complexes will be shown elsewhere.

### Specific interactions between VDR and VD3 derivatives

**Fig. 4:**
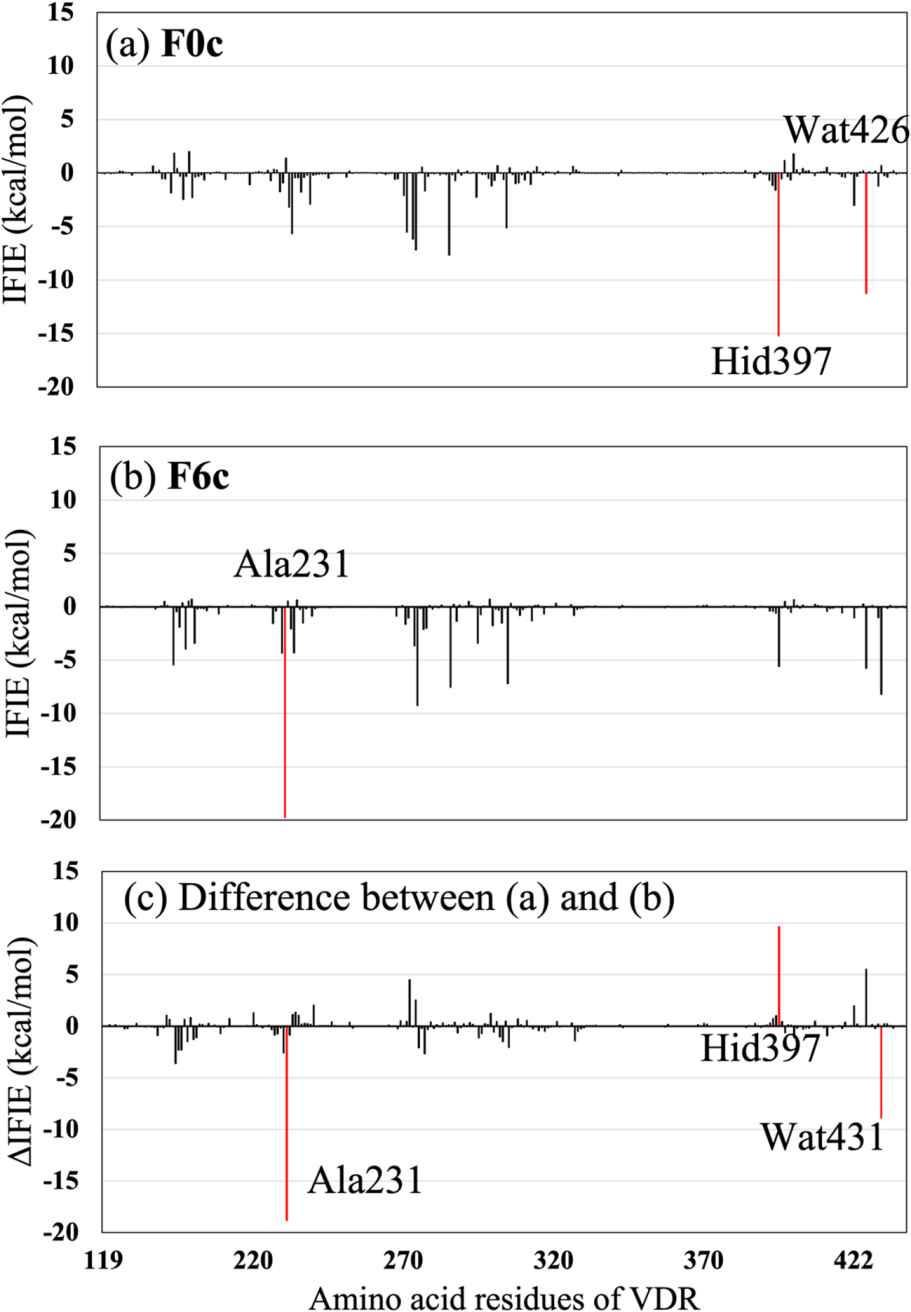
IFIEs evaluated using the present FMO method between each amino acid residue of VDR and VD3 derivatives: (a) **F0c**, (b) **F6c**, and (c) difference in IFIEs between (a) and (b). Red bars in (a) and (b) indicate the residues with strong attractive IFIEs whose size is larger than 10 kcal/mol. The red bars in (c) indicate the residues with significant differences in IFIEs whose size is larger than 6 kcal/mol

To clarify why introducing six fluorine atoms into **F0c** significantly increases its binding affinity to VDR, the IFIEs between the amino acid residues of VDR and each compound were analyzed using FMO calculations. The evaluated IFIEs for **F0c** and **F6c** were compared in Fig. 4. Negative ∆IFIEs in Fig. 4c indicate the amino acid residues or water molecules interacting more strongly with **F6c** compared with **F0c**. In contrast, positive ∆IFIEs suggest that certain amino acid residues interact more strongly with **F0c**. These changes in IFIEs were achieved by substituting six hydrogen atoms of the two CH_3_ groups at the end of **F0c** with fluorine atoms. As indicated in Fig. 4c, the substitution weakened the attractive interaction between **F0c** and Hid397 by about 10 kcal/mol, while this substitution enhanced the attractive interaction with Ala231 by about 19 kcal/mol. The interaction with a water molecule (Wat431) was also enhanced. As a result, the size of total IFIE between **F6c** and VDR became about 15 kcal/mol larger than that of **F0c** as shown in Table 1.

**Fig. 5:**
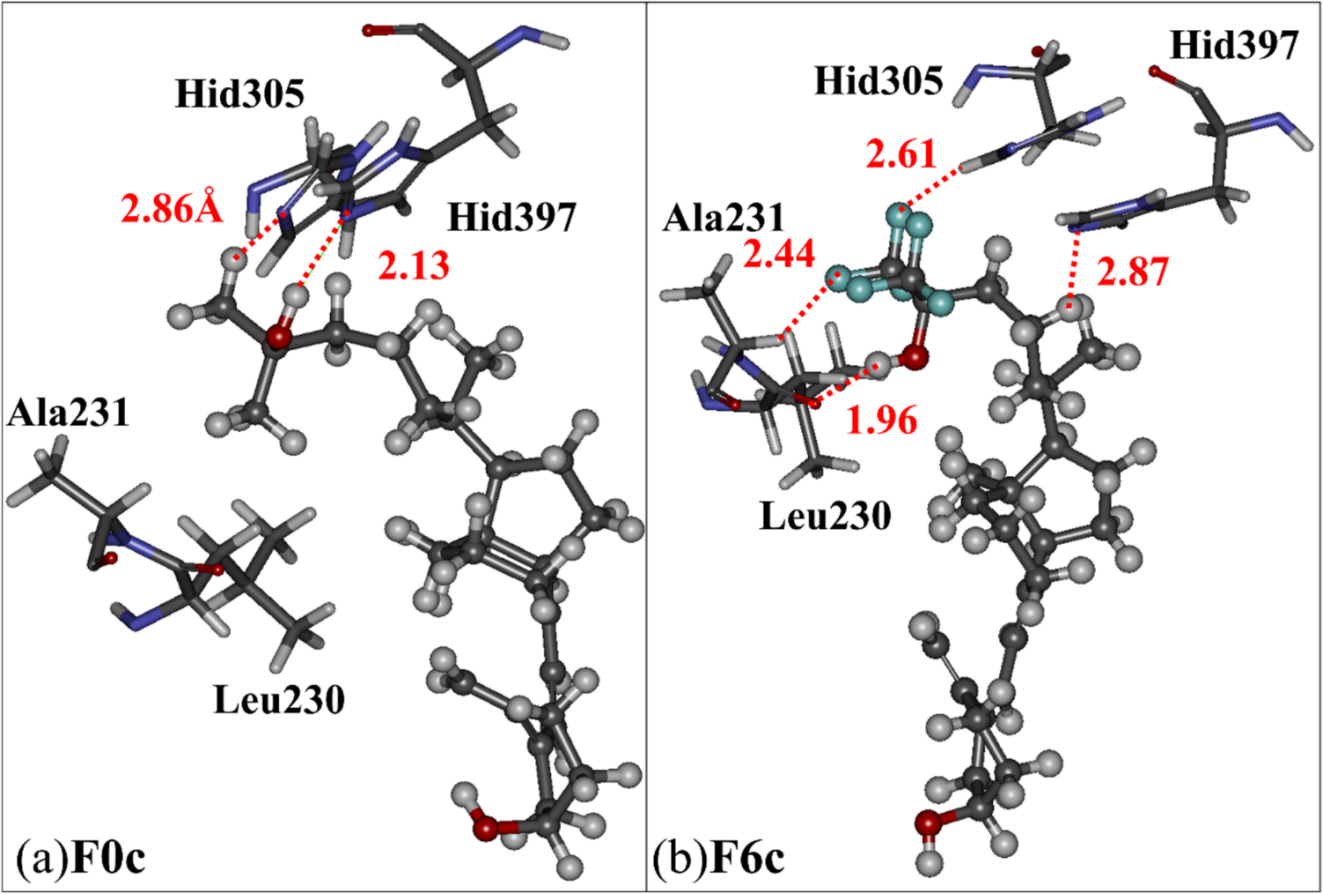
Interaction structures between critical VDR residues and compounds (ball & stick model) ((a) **F0c** and (b) **F6c**) in the VDR − compound complexes. Red lines indicate the distances between atoms of the compound and VDR residue. The present FMO calculations indicate that hydrogen atoms of the terminal CH_3_ groups of **F0c** have + 0.02 ~  + 0.14 charges, while F atoms (light blue in (b)) of the terminal CF_3_ groups of **F6c** have − 0.18 ~  − 0.22 charges

To clarify the cause of these changes in IFIEs, we compared the interaction structures between **F0c** or **F6c** and their surrounding VDR residues. As shown in Fig. 5a, the OH group of **F0c** forms a hydrogen bond with the nitrogen atom of the imidazole ring of the Hid397 side chain at 2.13 Å, resulting in a strong, attractive interaction between **F0c** and Hid397. In contrast, the terminal CH_3_ group of **F0c** is located at 2.86 Å from Hid305, indicating no strong attractive interaction between the terminal CH_3_ group and the other VDR residues.

In the complex of **F6c** and VDR, as shown in Fig. 5b, the substitution of all hydrogen atoms of the terminal two CH_3_ groups with fluorine atoms causes an electrostatic attraction between one of the fluorine atoms with negative charge (–0.18 ~ –0.22) and the Cα hydrogen atom of Ala231, bringing them closer together at 2.44 Å and significantly increasing the size of the attractive IFIE between **F6c** and Ala231, as shown in Figs. 4b and 4c. Furthermore, the fluorine substitution causes the terminal part of **F6c** to rotate, resulting in a significant change in the orientation of the terminal OH group, and consequently, the disappearance of the hydrogen bond between **F0c** and Hid397. Therefore, the present ab initio FMO calculations revealed that the introduction of six fluorine atoms into the two CH_3_ groups of **F0c** significantly changes the electrostatic interactions between **F0c** and the surrounding VDR residues to cause the rotation of the terminal group of two CF_3_ and OH of **F6c**, as shown in Fig. 5b.

**Fig. 6:**
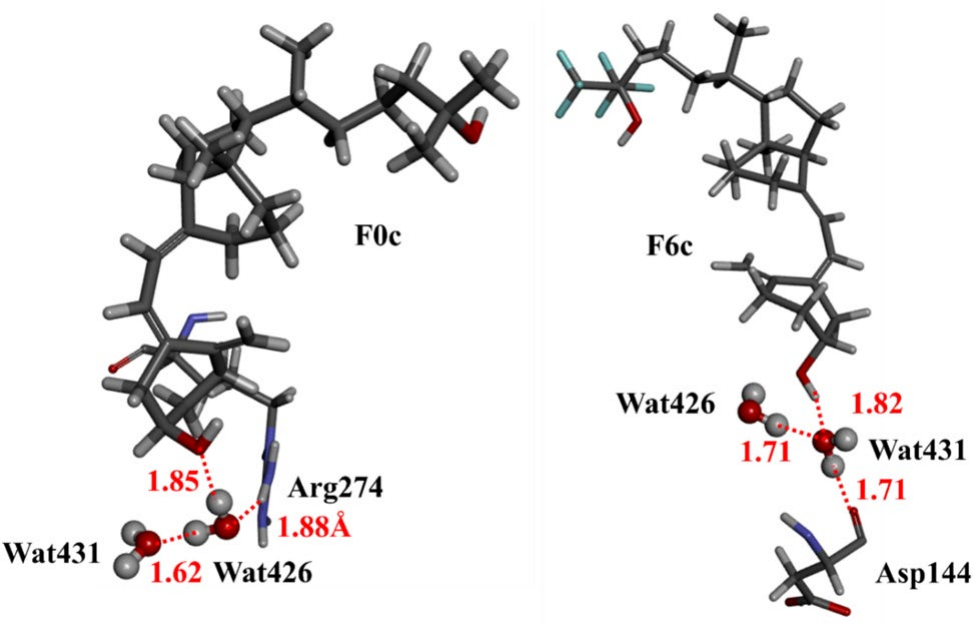
Hydrogen-bonding interactions between water molecules (Wat426 and Wat431 shown by ball & stick models), VDR residues and **F0c**/**F6c** compound in the VDR − compound complexes. Red lines indicate the distances of hydrogen bonds between water molecules, VDR residues, and compound. Wat426 and Wat431 act as bridging water molecules between VDR residue and compound in the VDR − **F0c** and VDR − **F6c** complexes, respectively

The IFIE graphs shown in Fig. 4 also indicate the contribution of water molecules (Wat426 and Wat431) to the interactions between VDR residues and **F0c**/**F6c**. To elucidate the specific interactions between these water molecules and VDR residues as well as **F0c**/**F6c**, we analyzed the hydrogen bonding interactions around these water molecules in the VDR–**F0c** and VDR–**F6c** complexes. As shown in Fig. 6, Wat426 forms hydrogen bonds with both Arg274 and **F0c** in the VDR–**F0c** complex, while Wat431 forms hydrogen bonds with both Asp144 and **F6c** in the VDR–**F6c** complex. Therefore, it is revealed that Wat426 and Wat431 can act as a bridging water molecule between VDR residue and **F0c**/**F6c**, in the VDR–compound complexes, respectively.

Next, to clarify why introducing two fluorine atoms into **F0c** resulted in a minor change (0.95-fold decrease) in its binding affinity to VDR, the IFIEs between **F2c** and the VDR residues were investigated using ab initio FMO calculations. In Figure S2c of SI, negative ∆IFIEs indicate amino acid residues that interact more strongly with **F2c** compared with **F0c**, while positive ∆IFIEs indicate residues with stronger interaction with **F0c**. The substitution of two hydrogen atoms in the terminal CH_3_ groups of **F0c** with fluorine atoms weakened the attractive interaction with Hid397 by about 12 kcal/mol. Meanwhile, **F2c** had weak attractive interactions with many amino acid residues, as shown in Figure S2b, resulting in a total IFIE of only 4 kcal/mol less than **F0c**. Therefore, as shown in Figure S2, the present FMO calculations indicated minimal changes in the interactions between VDR and **F0c/F2c**, consistent with the observed minor reduction in binding affinity noted in previous experimental studies [9].

We also investigated the interaction structures between **F0c** or **F2c** and the surrounding VDR residues to clarify the effects of fluorine substitution. As shown in Figure S3 of SI, the terminal two CH_2_F and OH groups of **F2c** rotated significantly due to fluorine substitution, causing a notable change in the direction of the OH group. As a result, the attractive interaction between the OH group of **F0c** and Hid397 at 2.13 Å (Figure S3a) was significantly weakened by the two fluorine atoms substitution. One fluorine atom of the CH_2_F groups interacted with the side chain of Hid305 at 2.45 Å, while the other fluorine atom did not exhibit strong interactions with VDR residues. Consequently, the interaction structures between VDR residues and **F0c** and **F2c** were similar, as shown in Figure S3 of SI.

To clarify the reason for the 7.1-fold increase in the binding affinity of **F0c** to VDR upon introducing four fluorine atoms, we analyzed the IFIEs between **F4c** and VDR residues using ab initio FMO calculations. In Figure S4c of SI, negative ∆IFIEs indicate residues that interact more strongly with **F4c**, while positive ∆IFIEs indicate residues that interact more strongly with **F0c**. By substituting two H atoms in each of the two terminal CH_3_ groups of **F0c** with fluorine atoms, the attractive interaction with Hid397 was weakened by about 11 kcal/mol. In contrast, the attractive interaction with Hid305 was enhanced by about 14 kcal/mol, resulting in a larger size of total IFIE for **F4c** than for **F0c** as indicated in Table 1.

In the VDR–**F4c** complex, the terminal OH group of **F4c** formed a strong hydrogen bond with the nitrogen atom of the imidazole ring of the Hid305 side chain at 1.81 Å, as shown in Figure S5b of SI. This strong attractive interaction is likely to be a main reason for the fact that the total IFIE between **F4c** and VDR is 5 kcal/mol higher than that for **F0c**. This finding is comparable to the trend of binding affinities obtained by the previous experiments [9] as shown in Table 1 and Fig. 2.

Finally, to clarify the effect of the change in configuration of the fluorine substituted compounds on their interactions with VDR, we compared their IFIEs and interaction structures for **F0** and **F0c**, as well as for **F6** and **F6c**. The results comparing **F0** and **F0c** are shown in Figures S6 and S7 of SI, where negative ∆IFIEs in Figure S6c indicate amino acid residues that interact more strongly with **F0c**, and positive ∆IFIEs indicate amino acid residues that interact more strongly with **F0**. By the change of configuration of **F0**, the attractive interactions between **F0** and Hid397, Water426, and Met272 were strengthened, while that with Ser278 was weakened.

To elucidate the reason for these changes in IFIEs, in Figure S7 of SI, we compared the interaction structures between **F0/F0c** and the surrounding VDR residues in the complex structure of VDR containing **F0** or **F0c**. The OH group at the 25th site of **F0** and **F0c** formed a hydrogen bond with the nitrogen atom of the imidazole ring of the Hid397 side chain. Due to the change in configuration of **F0**, the orientation of the OH group changed significantly, and the distance between the OH group and the Hid397 side chain was shortened from 2.83 Å to 2.13 Å. As a result, the attractive interaction between **F0** and Hid397 was enhanced due to the change of configuration, as shown in Figure S6c of SI. In contrast, the other OH group at the C-3 site of **F0** exhibited a different effect by the configuration change, as shown in the lower part of Figures S7a and S7b. The OH group of **F0** formed a hydrogen bond (1.76 Å) with the Ser278 side chain, while that of **F0c** formed a hydrogen bond with Water426. Accordingly, it was elucidated that the interactions between the OH groups of **F0** and VDR residues change significantly due to the change in the configuration of **F0**.

In a similar manner, we compared IFIEs and interaction structures for the VDR complexes with **F6** or **F6c** in Figures S8 and S9 of SI, respectively. In Figure S8c, negative ∆IFIEs indicate amino acid residues that interact more strongly with **F6c**, while positive ∆IFIEs indicate amino acid residues that interact more strongly with **F6**. As shown in Figures S8a and S8b of SI, both **F6** and **F6c** exhibited strong attractive interactions with Ala231. However, as shown in Figure S9 of SI, the shortest distance between Ala231 and the fluorine atom at the CF_3_ group varied remarkably (2.78 Å for **F6** and 2.44 Å for **F6c**), depending on the configuration of **F6**. As a result, **F6c** exhibited a more significant attractive interaction (–20 kcal/mol) with Ala231 compared with **F6**, as illustrated in Figures S8a and S8b of SI. Furthermore, as shown in Figure S9 of SI, the orientation of the OH group associated with the cyclohexane group at the C-3 site of **F6** varied markedly due to the difference in the configuration. This change caused significant alterations in the interactions between **F6** and both Ser278 and surrounding water molecules, as depicted in Figure S9 of SI.

As shown in Figures S6–S9 of SI, the specific interactions between VDR residues and **F0** and **F6** compounds were affected significantly by the change in the configuration of the compounds. To understand this effect more detail, we compared the structures of **F0** and **F6** in their complexes with VDR to those of **F0c** and **F6c**. As depicted in Figure S10 of SI, the conformation of the terminal region with two CH_3_ and OH groups varied significantly depending on the configuration of the compounds. This variation caused the change in the interactions between each compound and VDR residues. According to the results presented in Fig. 2, the evaluated total IFIEs for the **F0c**, **F2c**, **F4c**, and **F6c** compounds correlate well with the trend of binding affinities observed in the previous experiment [9]. Therefore, the configuration of **F0c**, **F2c**, **F4c**, and **F6c** appears to be more representative than that of **F0**, **F2**, **F4**, and **F6**. Based on **F6c**, which has the highest total IFIE among the **F0c** based compounds, new compounds that can bind more strongly to VDR will be proposed upon analysis of their binding properties to VDR.

## Conclusions

To elucidate how the fluorine substitution in VD3 derivatives influences their binding properties to VDR, we investigated the specific interactions between VDR and the fluorine substituted VD3 derivatives. Our approach included molecular simulations based on MM optimizations, MD simulations, and ab initio FMO calculations. The main findings are summarized as follows.

1. The change of configuration in the fluorine substituted derivatives may affect their interactions with VDR. The evaluated total IFIEs between VDR residues and each of the derivatives (**F0c**, **F2c**, **F4c**, and **F6c** shown in Fig. 1) are comparable to the trend in binding affinities observed in the previous experiment [9].
2. The introduction of six fluorine atoms into the two CH_3_ groups of **F0c** alters the charge distribution within **F0c**, modifying the electrostatic interactions between **F0c** and the surrounding VDR residues. Consequently, the terminal portion of the two CH_3_ and OH groups rotates significantly, forming a hydrogen bond between **F6c** and the oxygen atom of the backbone between Ala231 and Leu230, as shown in Fig. 5b.
3. The substitutions of two or four fluorine atoms within **F0c** have not so significant effect on the interactions between **F0c** and VDR residues as that for the VDR–**F6c** complex.

In our future study, we will extend the present molecular simulations for other types of VD3 derivatives and explore more widely novel VD3 derivatives as candidate compounds for VDR inhibitors.

## Supplementary Information

Below is the link to the electronic supplementary material.

Supplementary file1 (DOCX 2870 KB)

## Data Availability

All data simulated or analyzed during this study are included in both this article and the supporting information.
