# Supplementary material for: Specific interactions between fluorinated vitamin-D3 derivatives and vitamin-D receptor: molecular mechanics and ab initio fragment molecular orbital calculations
Source: J Mol Model. 2026 Jan 14;32(2):41. doi: 10.1007/s00894-025-06623-1 (PMC12804298; doi:10.1007/s00894-025-06623-1)
Supplement: Supplementary file 1 — Supplementary file1 (DOCX 2870 KB) [file 894_2025_6623_MOESM1_ESM.docx]

**Supplementary information of**

**“Specific interactions between fluorinated vitamin-D_3_ derivatives and vitamin-D receptor: molecular mechanics and *ab initio* fragment molecular orbital calculations”**

Masayuki Yuguchi^1^, Shuta Takenaka^1^, Chisato Nakatani^1^, Yoshinobu Nagura^1^, Haruna Sabishiro^1^, Nagomi Chimura^1^, Atsushi Kittaka^2^, Midori Takimoto-Kamimura^3^, and Noriyuki Kurita^1,^ *

^1^ Department of **Computer Science and Engineering**, Toyohashi University of Technology, Tempaku-cho, Toyohashi, Aichi, 441-8580, Japan

*^2^ Faculty of Pharmaceutical Sciences, Teikyo University,
2-11-1 Kaga, Itabashi, Tokyo 173-8605, Japan*

^3^ *Quantum-Structural Life Science Laboratories, CBI Research Institute,
Kyowa Create Daiichi build. 3F, 3-11-1 Shibaura Minato, Tokyo, 108-2234, Japan.*

***Corresponding author**

Noriyuki Kurita, Associate Professor, Ph.D.

Department of **Computer Science and Engineering,**

Toyohashi University of Technology,

Tempaku-cho, Toyohashi, Aichi, 441-8580, Japan

E-mail: [kurita@cs.tut.ac.jp](mailto:kurita@cs.tut.ac.jp)

Tel. & Fax.: +81-532-44-6875

**Contents**

Table S1 Protonation states of His residues of VDR in VDR−compound complex assigned in the present study.

Table S2 Total energies (TEs; kcal/mol) evaluated using the present FMO method of VDR−**F0**/**F6** complexes for each His protonation state. Relative values of TE compared with that of the most stable Hid/Hid protonation state are listed. Hid/Hid means His305 and His397 have Hid and Hid states, respectively.

Table S3 Difference in total energies (kcal/mol) evaluated using the present FMO method for compounds (**F0c**, **F2c**, **F4c**, **F6c**) compared with those (**F0**, **F2**, **F4**, **F6**) having different diastereomeric forms as shown in Figure 1.

Figure S1 Possible protonation states of histidine residue.

Figure S2 IFIEs evaluated using the present FMO method between each amino acid residue of VDR and VD3 derivatives: (a) **F0c**, (b) **F2c**, and (c) difference in IFIEs between (a) and (b). Red bars in (a) and (b) indicate the residues with strong attractive IFIEs higher than 10 kcal/mol. The red bars in (c) indicate the residues with significant differences in IFIEs whose size is larger than 10 kcal/mol.

Figure S3 Interaction structures between critical VDR residues and compounds ((a) **F0c** and (b) **F2c**) in the VDR−compound complexes. Red lines indicate the distances between atoms of the compound and VDR residue. The present FMO calculations indicate that hydrogen atoms of the terminal CH_3_ groups of **F0c** have +0.02 ~ +0.14 charges, while F atoms (light blue in (b)) of the terminal CF groups of **F2c** have −0.26 charges.

Figure S4 IFIEs evaluated using the present FMO method between each amino acid residue of VDR and VD3 derivatives: (a) **F0c**, (b) **F4c**, and (c) difference in IFIEs between (a) and (b). Red bars in (a) and (b) indicate the residues with strong attractive IFIEs higher than 10 kcal/mol. The red bars in (c) indicate the residues with significant differences in IFIEs whose size is larger than 10 kcal/mol.

Figure S5 Interaction structures between critical VDR residues and compounds ((a) **F0c** and (b) **F4c**) in the VDR−compound complexes. Red lines indicate the distances between atoms of the compound and VDR residue. The present FMO calculations indicate that hydrogen atoms of the terminal CH_3_ groups of **F0c** have +0.02 ~ +0.14 charges, while F atoms (light blue in (b)) of the terminal CF_2_ groups of **F4c** have −0.20 ~ −0.29 charges.

Figure S6 IFIEs evaluated using the present FMO method between each amino acid residue of VDR and VD3 derivatives: (a) **F0c**, (b) **F0**, and (c) difference in IFIEs between (a) and (b). Red bars in (a) and (b) indicate the residues with strong attractive IFIEs whose size is larger than 10 kcal/mol. The red bars in (c) indicate the residues with significant differences in IFIEs whose size is larger than 5 kcal/mol.

Figure S7 Interaction structures between critical VDR residues and compounds ((a) **F0c** and (b) **F0**) in the VDR−compound complexes. Red lines indicate the distances between atoms of the compound and VDR residue. The present FMO calculations indicate that hydrogen atoms of the terminal CH_3_ groups of **F0c** have +0.02 ~ +0.14 charges, while those of **F0** have +0.02 ~ +0.14 charges.

Figure S8 IFIEs evaluated using the present FMO method between each amino acid residue of VDR and VD3 derivatives: (a) **F6c**, (b) **F6**, and (c) difference in IFIEs between (a) and (b). Red bars in (a) and (b) indicate the residues with strong attractive IFIEs whose size is larger than 10 kcal/mol. The red bars in (c) indicate the residues with significant differences in IFIEs whose size is larger than 5 kcal/mol.

Figure S9 Interaction structures between critical VDR residues and compounds ((a) **F6c** and (b) **F6**) in the VDR−compound complexes. Red lines indicate the distances between atoms of the compound and VDR residue. The present FMO calculations indicate that F atoms of the terminal CF_3_ groups of **F6c** have +0.02 ~ +0.14 charges, while those of **F6** have +0.02 ~ +0.14 charges.

Figure S10 Optimized structures using MM method of (a) **F0** and **F6,** and (b) **F0c** and **F6c** in their complexes with VDR.

Table S1 Protonation states of His residues of VDR in VDR−compound complex assigned in the present study.

| His residue | pKa value | His protonation |
| --- | --- | --- |
| His139 | 2.97 | Hid (H-bond with Glu327) |
| His140 | 6.68 | Hip^+^ (outside of protein) |
| His229 | 4.89 | Hid (H-bond with Tyr295) |
| His305 | 4.38 | Hie or Hid |
| His326 | 6.01 | Hip^+^ (outside of protein) |
| His330 | 3.95 | Hid (H-bond with a crystal water) |
| His371 | 6.81 | Hip^+^ (outside of protein) |
| His377 | 6.20 | Hip^+^ (outside of protein) |
| His397 | 2.34 | Hie or Hid |

Table S2 Total energies (TEs; kcal/mol) evaluated using the present FMO method of VDR−**F0**/**F6** complexes for each His protonation state. Relative values of TE compared with that of the most stable Hid/Hid protonation state are listed. Hid/Hid means His305 and His397 have Hid and Hid states, respectively.


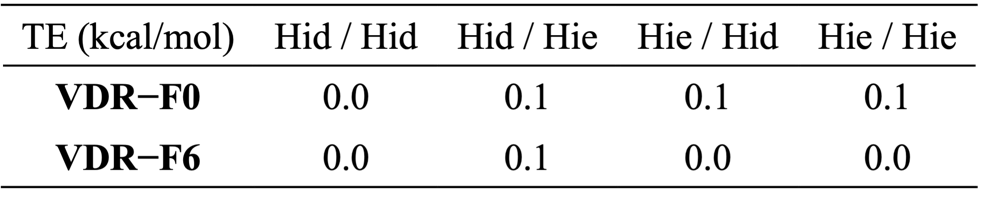


Table S3 Difference in total energies (kcal/mol) evaluated using the present FMO method for compounds (**F0c**, **F2c**, **F4c**, **F6c**) compared with those (**F0**, **F2**, **F4**, **F6**) having different diastereomeric forms as shown in Figure 1.


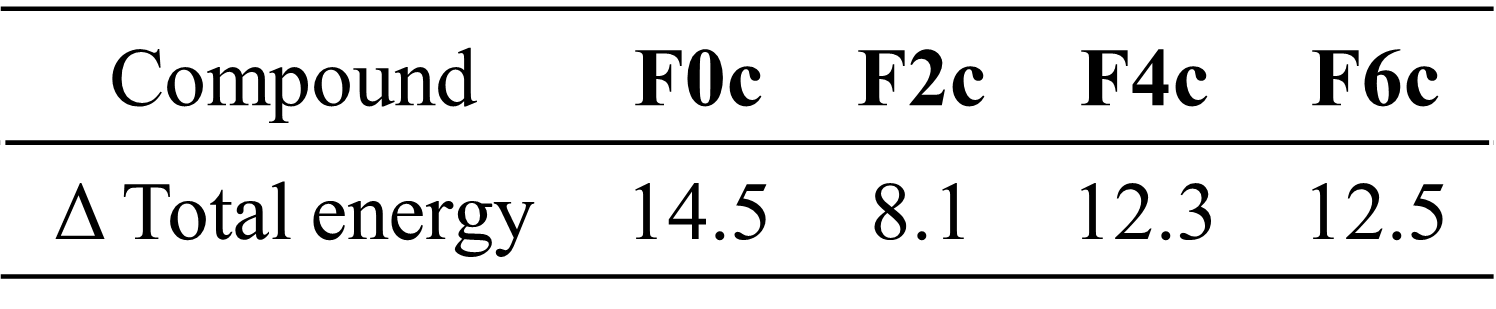


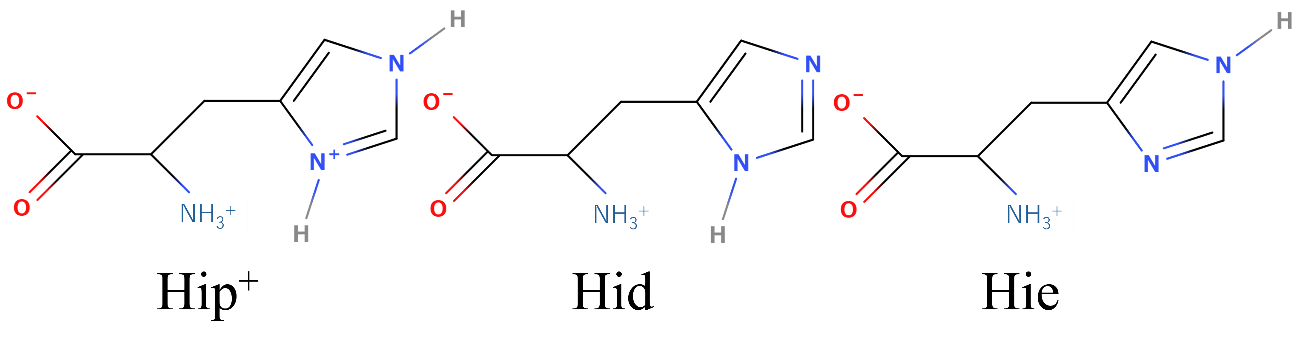


Figure S1 Possible protonation states of histidine residue.


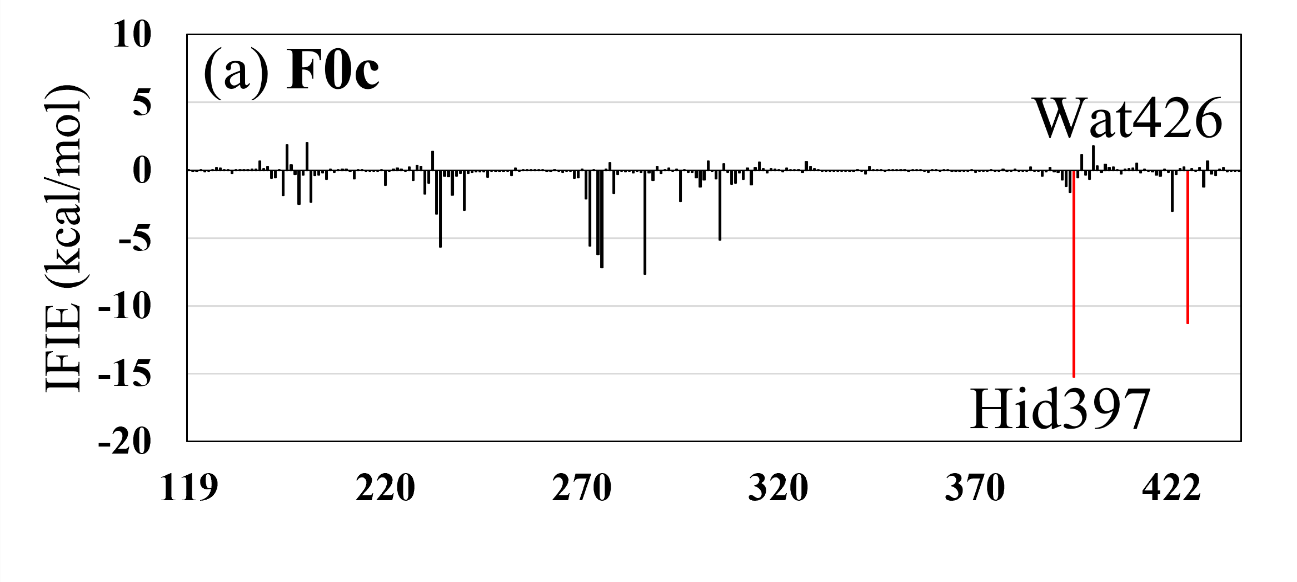

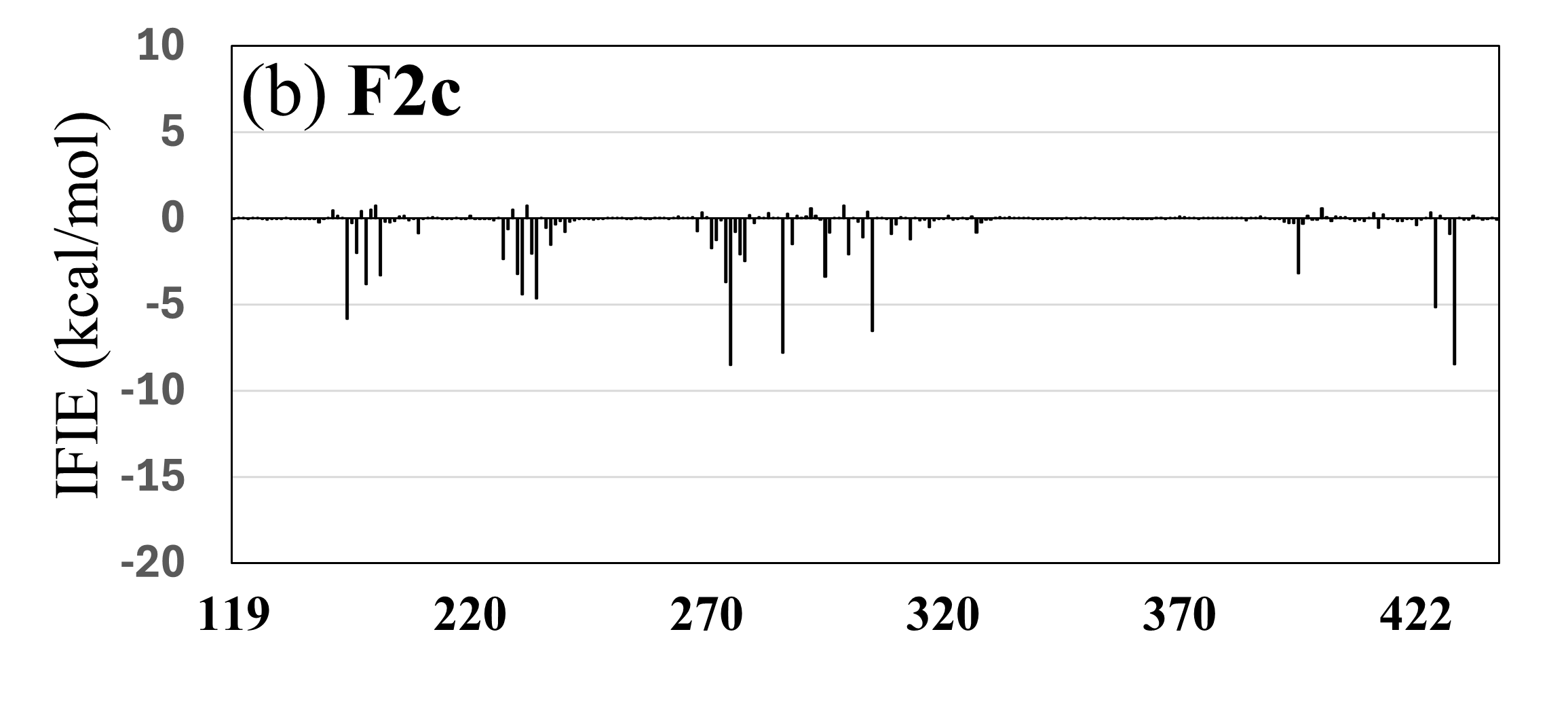


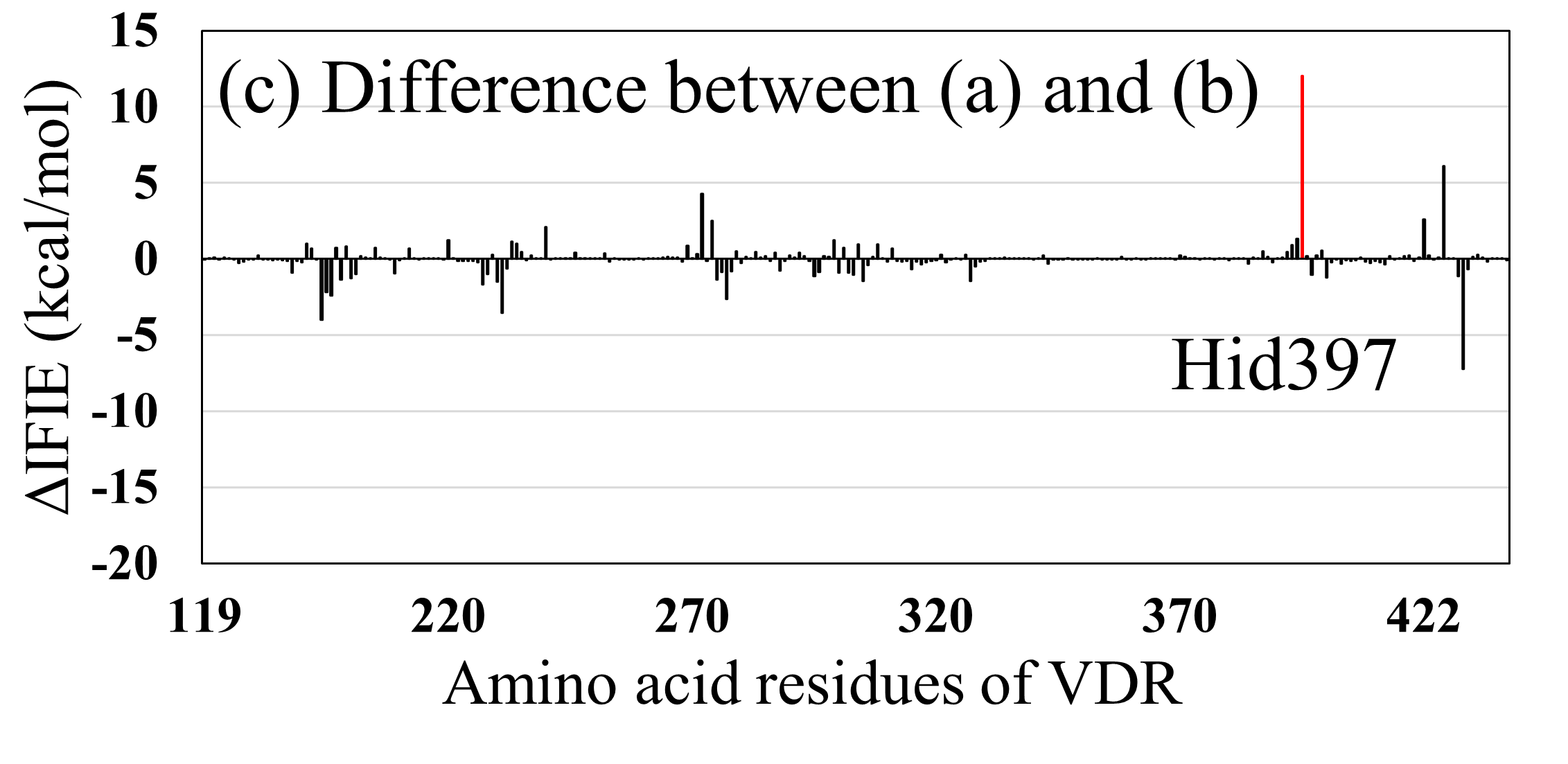


Figure S2 IFIEs evaluated using the present FMO method between each amino acid residue of VDR and VD3 derivatives: (a) **F0c**, (b) **F2c**, and (c) difference in IFIEs between (a) and (b). Red bars in (a) and (b) indicate the residues with strong attractive IFIEs higher than 10 kcal/mol. The red bars in (c) indicate the residues with significant differences in IFIEs whose size is larger than 10 kcal/mol.


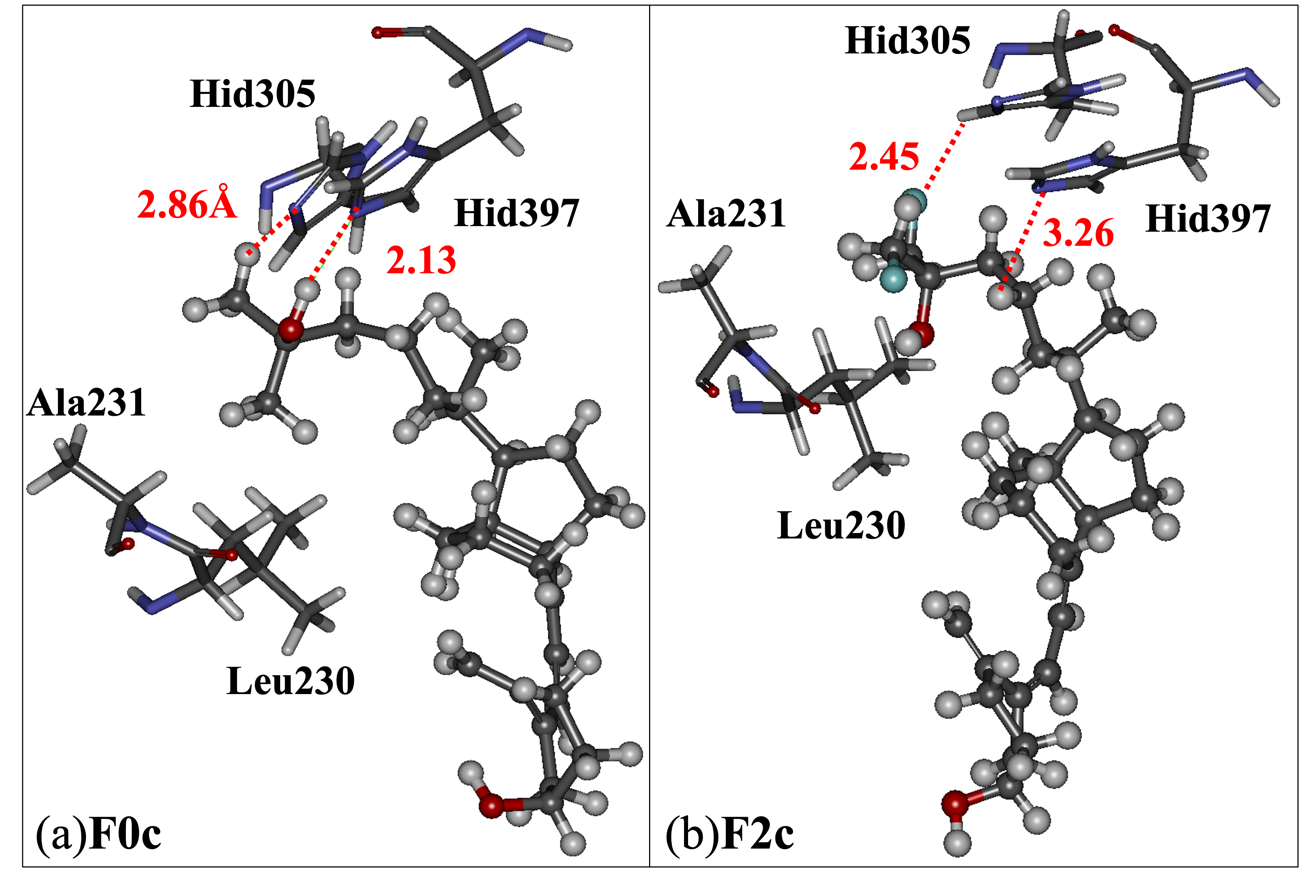


Figure S3 Interaction structures between critical VDR residues and compounds ((a) **F0c** and (b) **F2c**) in the VDR−compound complexes. Red lines indicate the distances between atoms of the compound and VDR residue. The present FMO calculations indicate that hydrogen atoms of the terminal CH_3_ groups of **F0c** have +0.02 ~ +0.14 charges, while F atoms (light blue in (b)) of the terminal CF groups of **F2c** have −0.26 charges.


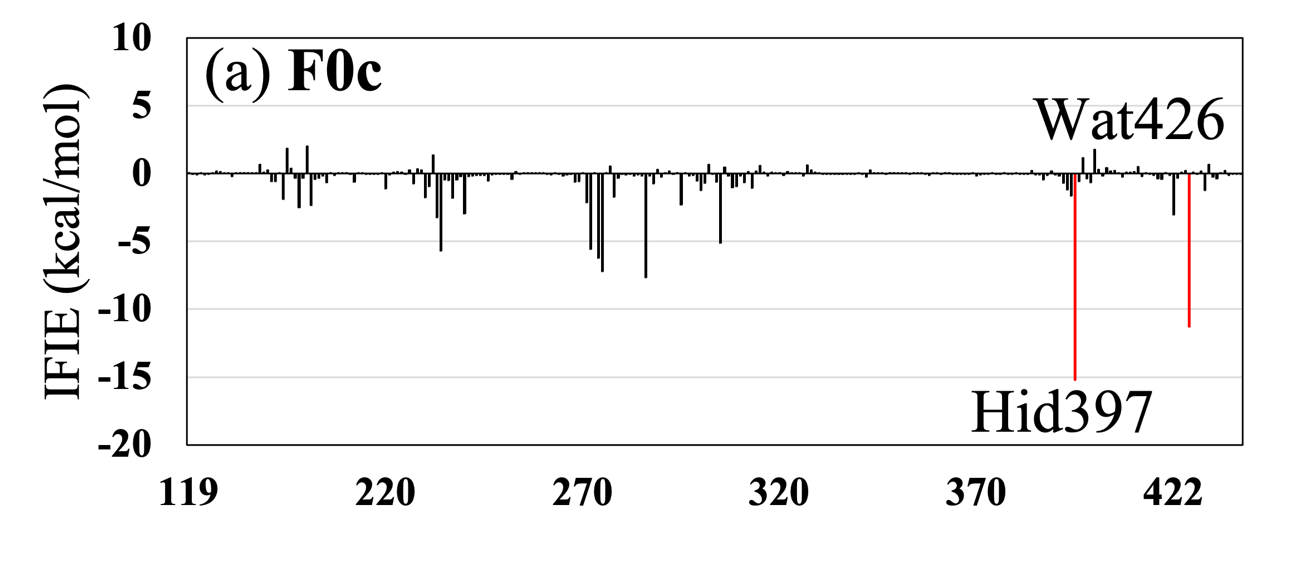


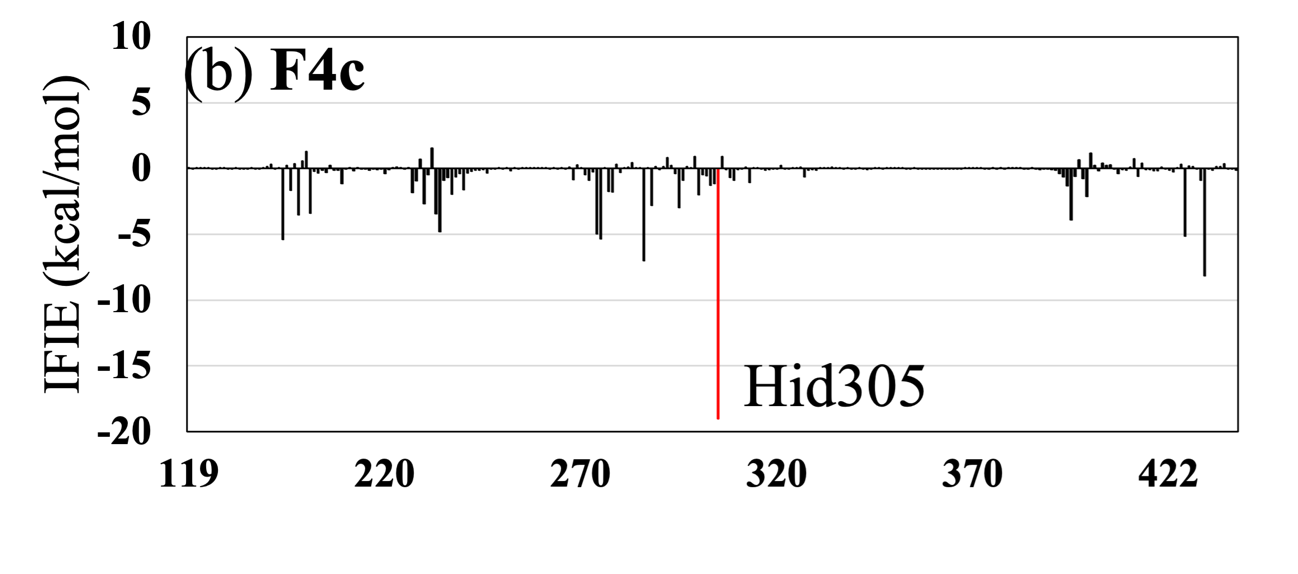


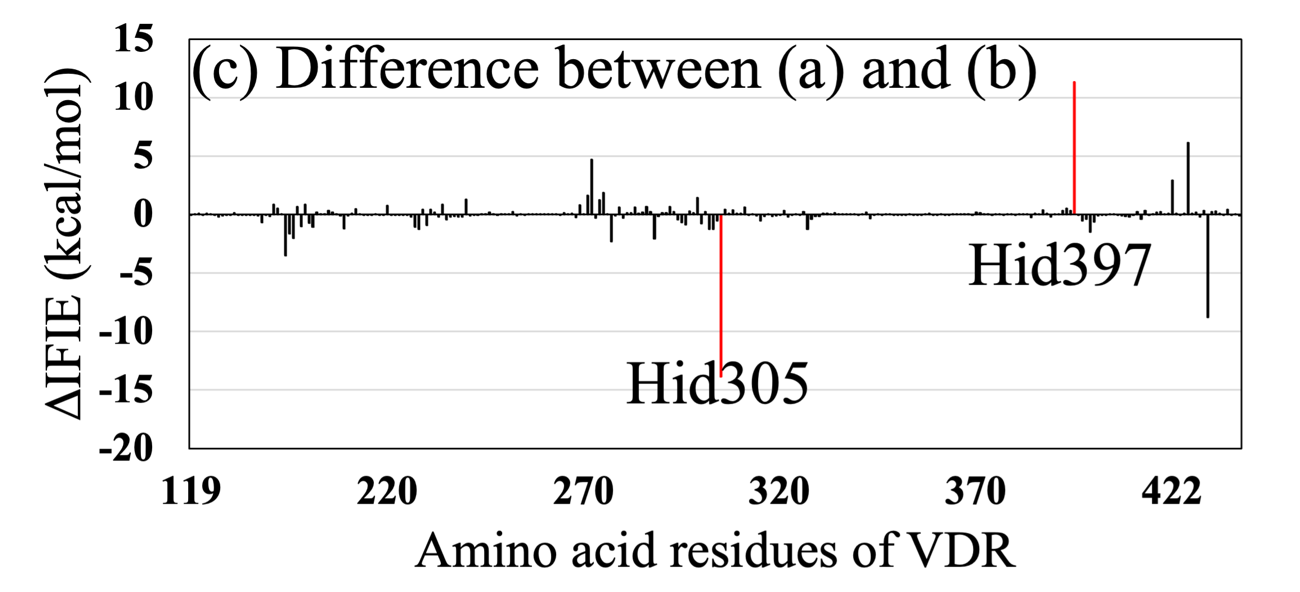


Figure S4 IFIEs evaluated using the present FMO method between each amino acid residue of VDR and VD3 derivatives: (a) **F0c**, (b) **F4c**, and (c) difference in IFIEs between (a) and (b). Red bars in (a) and (b) indicate the residues with strong attractive IFIEs higher than 10 kcal/mol. The red bars in (c) indicate the residues with significant differences in IFIEs whose size is larger than 10 kcal/mol.


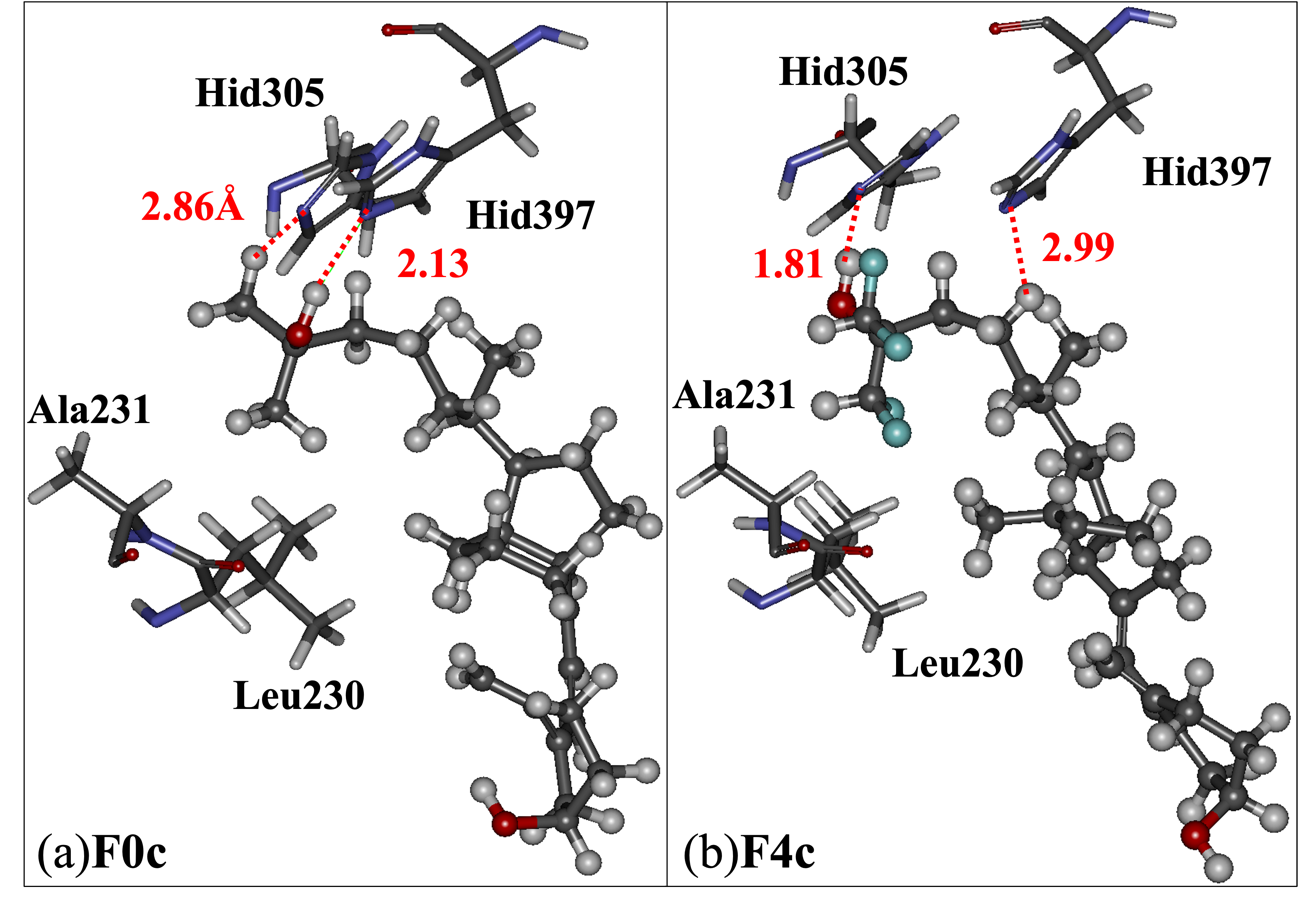


Figure S5 Interaction structures between critical VDR residues and compounds ((a) **F0c** and (b) **F4c**) in the VDR−compound complexes. Red lines indicate the distances between atoms of the compound and VDR residue. The present FMO calculations indicate that hydrogen atoms of the terminal CH_3_ groups of **F0c** have +0.02 ~ +0.14 charges, while F atoms (light blue in (b)) of the terminal CF_2_ groups of **F4c** have −0.20 ~ −0.29 charges.


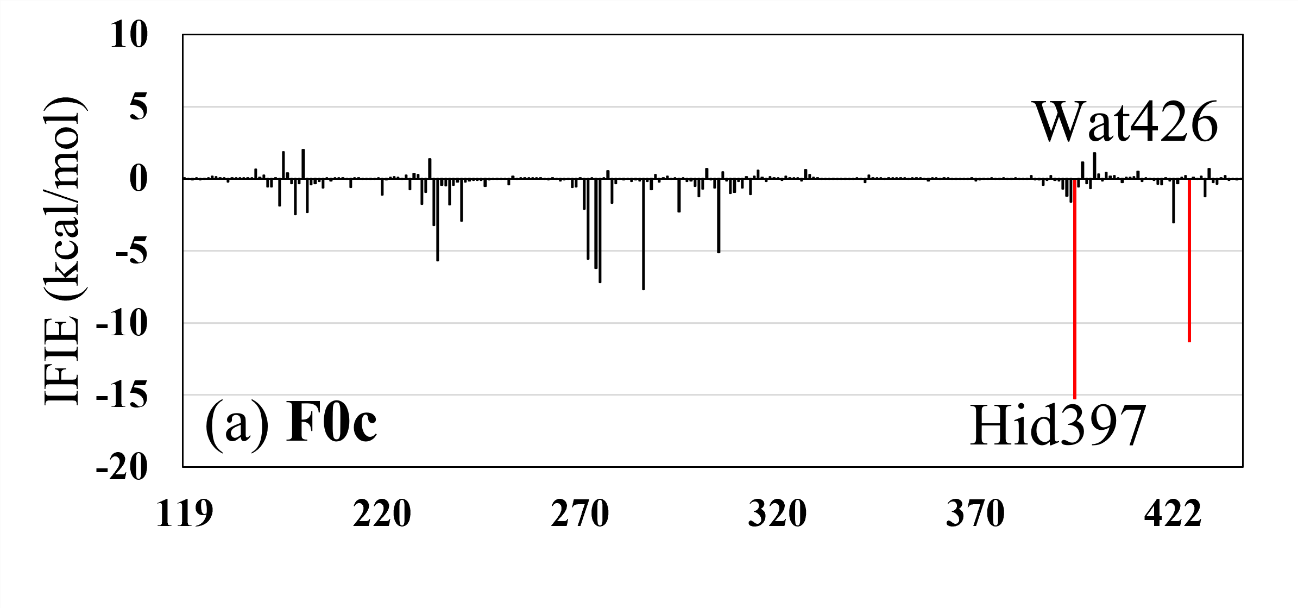

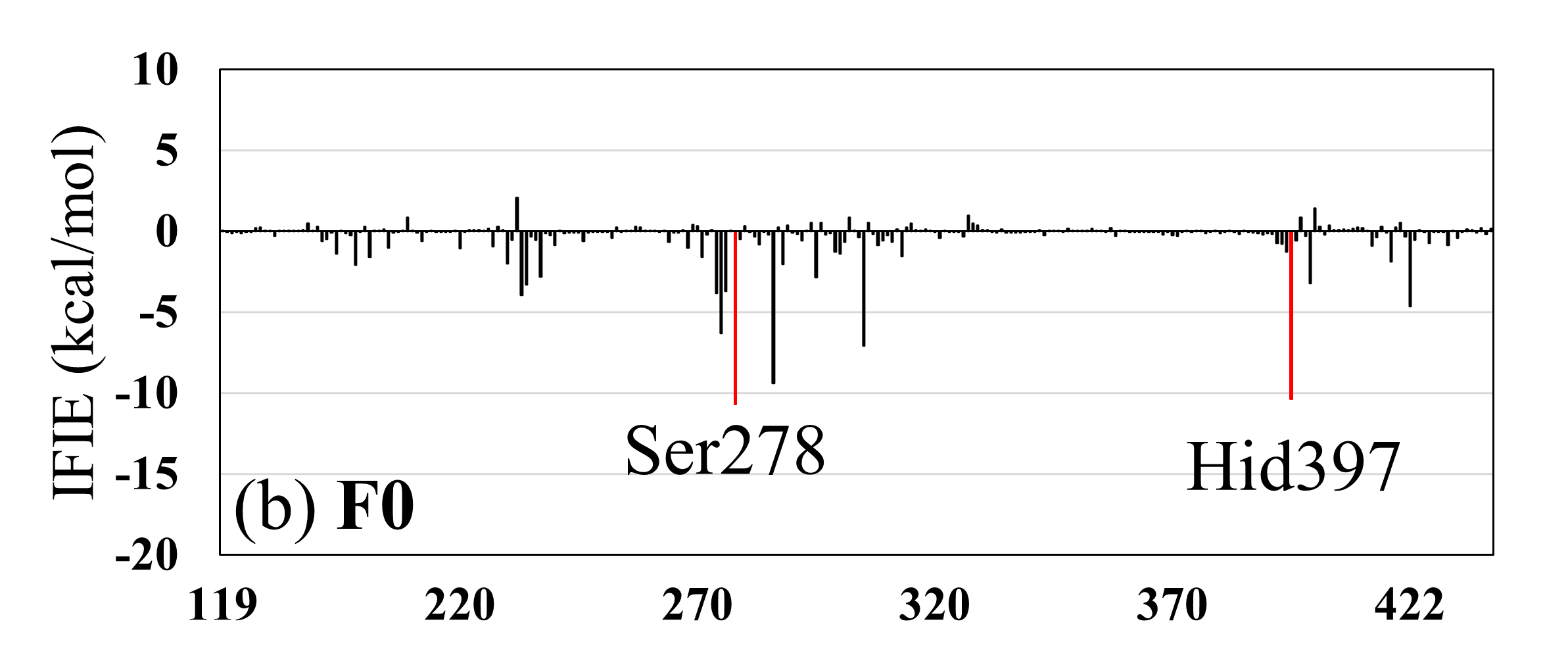


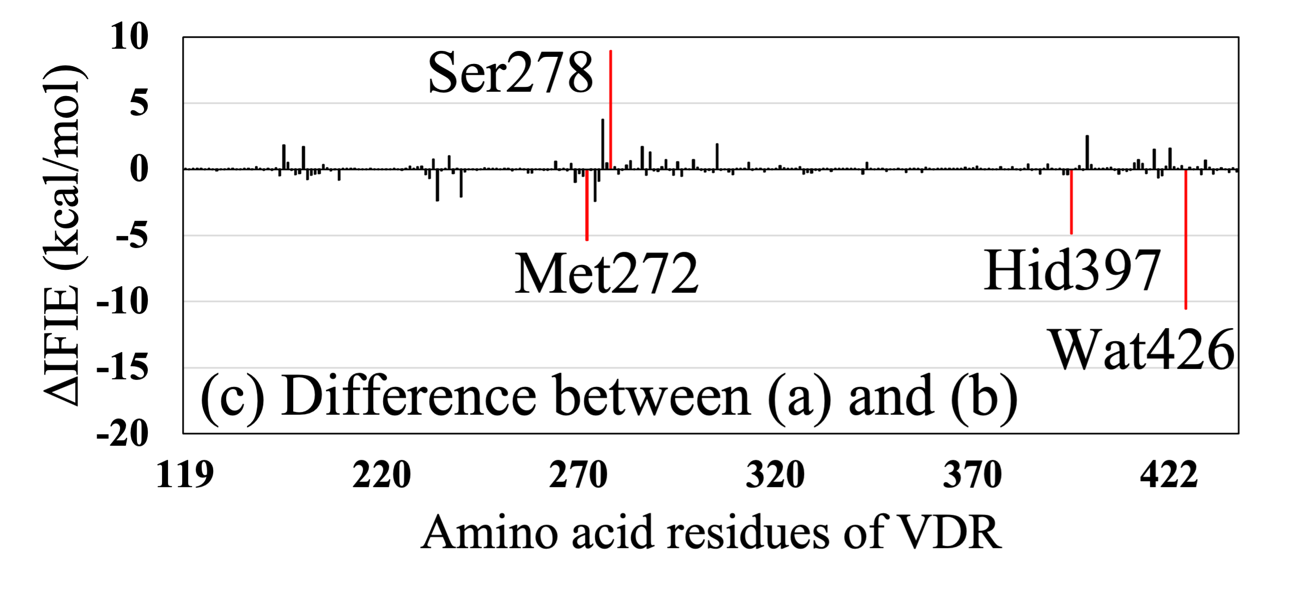


Figure S6 IFIEs evaluated using the present FMO method between each amino acid residue of VDR and VD3 derivatives: (a) **F0c**, (b) **F0**, and (c) difference in IFIEs between (a) and (b). Red bars in (a) and (b) indicate the residues with strong attractive IFIEs whose size is larger than 10 kcal/mol. The red bars in (c) indicate the residues with significant differences in IFIEs whose size is larger than 5 kcal/mol.


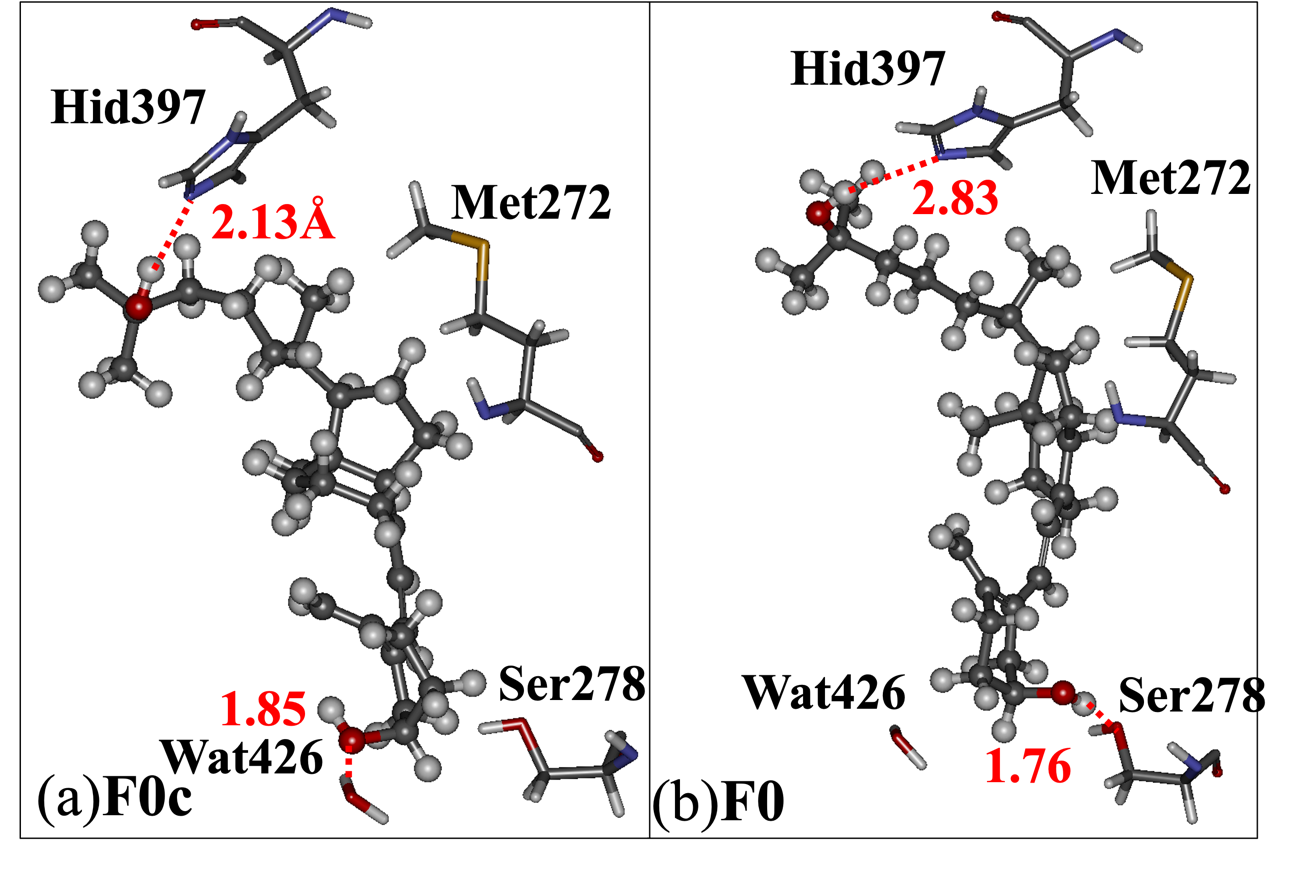


Figure S7 Interaction structures between critical VDR residues and compounds ((a) **F0c** and (b) **F0**) in the VDR−compound complexes. Red lines indicate the distances between atoms of the compound and VDR residue. The present FMO calculations indicate that hydrogen atoms of the terminal CH_3_ groups of **F0c** have +0.02 ~ +0.14 charges, while those of **F0** have +0.02 ~ +0.14 charges.


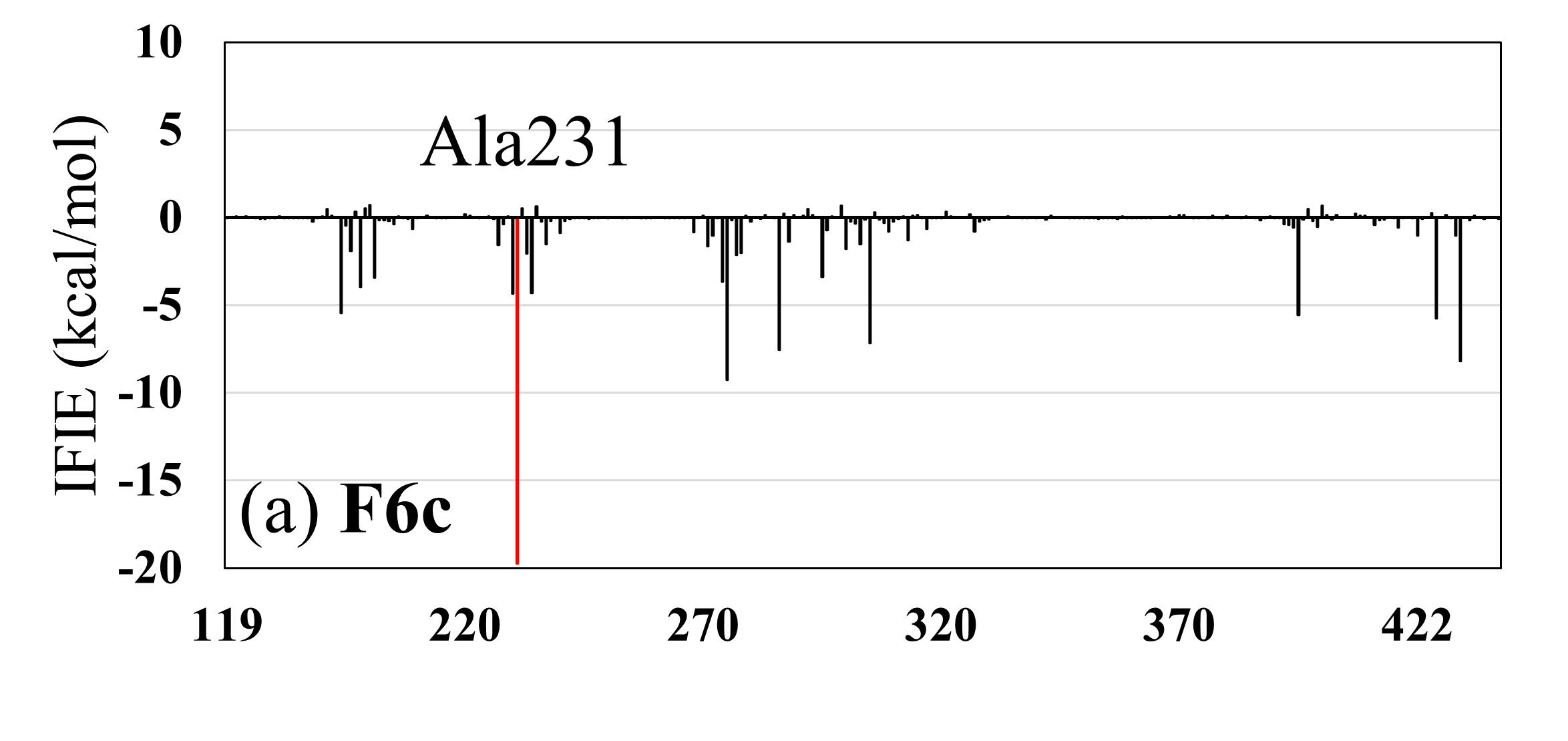


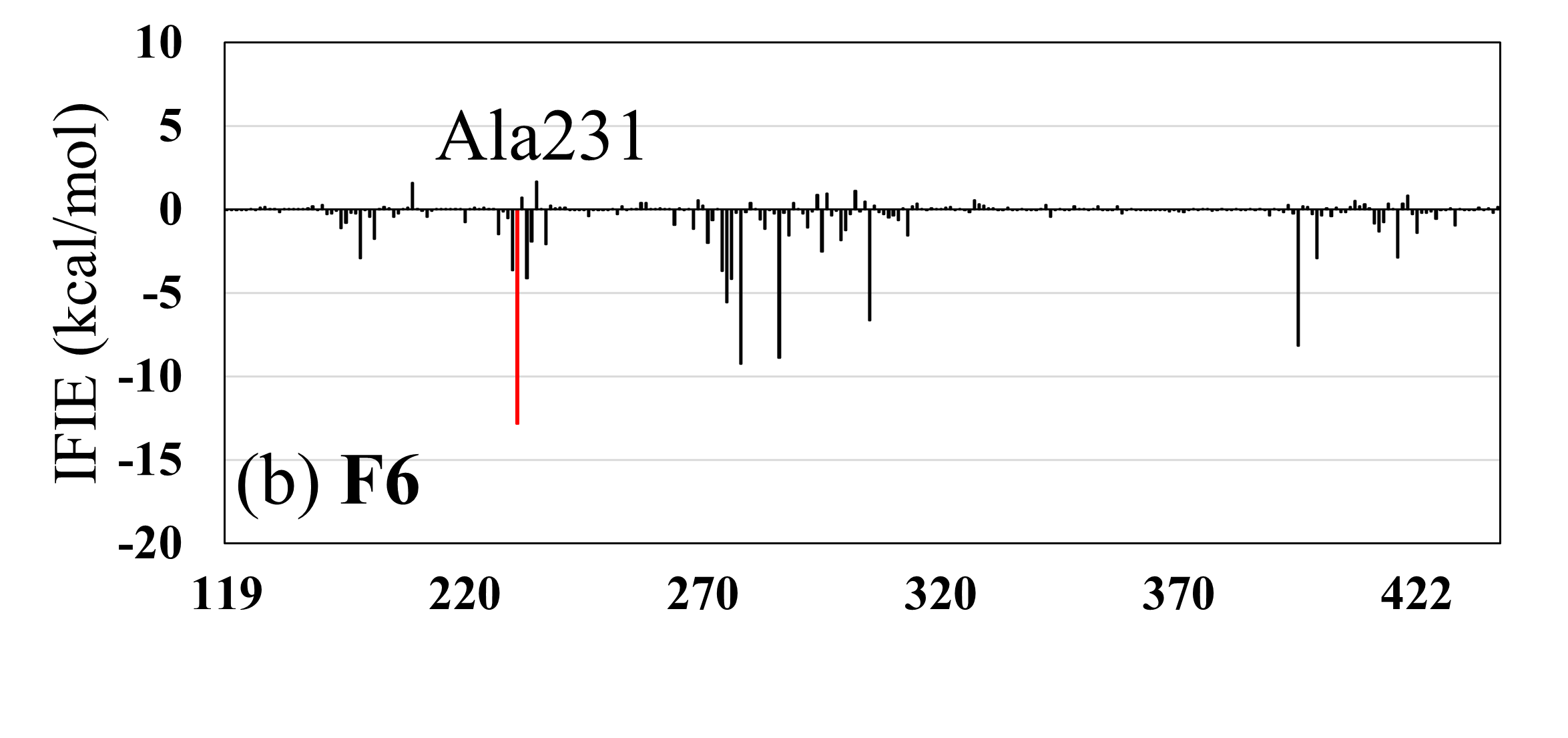


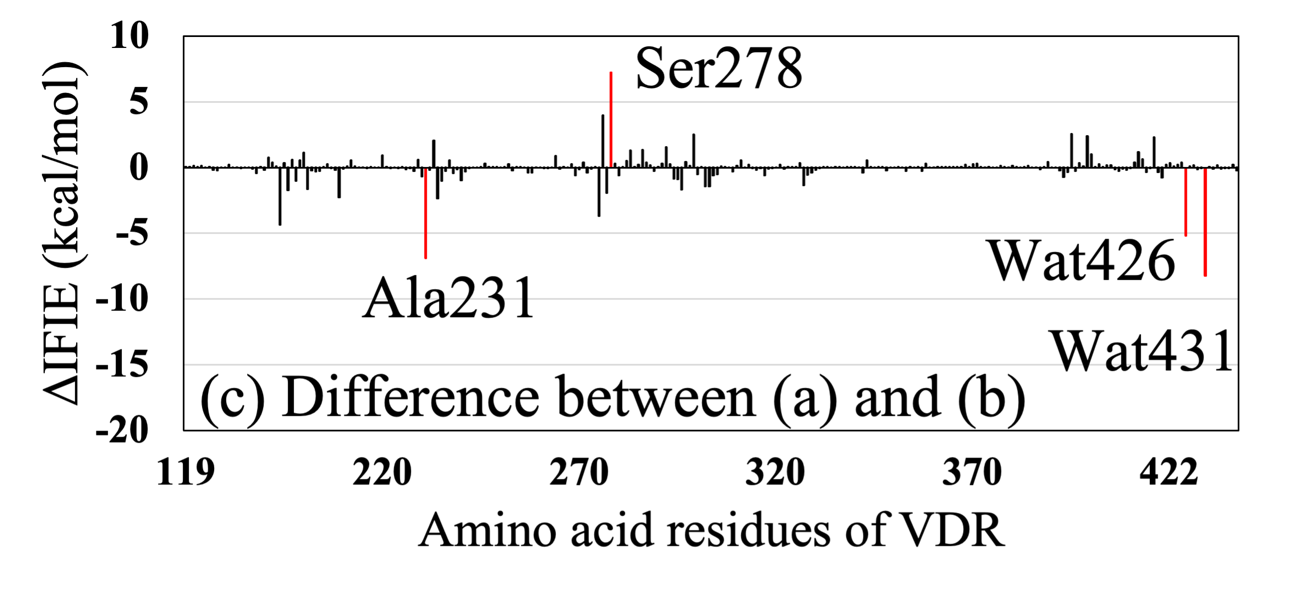


Figure S8 IFIEs evaluated using the present FMO method between each amino acid residue of VDR and VD3 derivatives: (a) **F6c**, (b) **F6**, and (c) difference in IFIEs between (a) and (b). Red bars in (a) and (b) indicate the residues with strong attractive IFIEs whose size is larger than 10 kcal/mol. The red bars in (c) indicate the residues with significant differences in IFIEs whose size is larger than 5 kcal/mol.


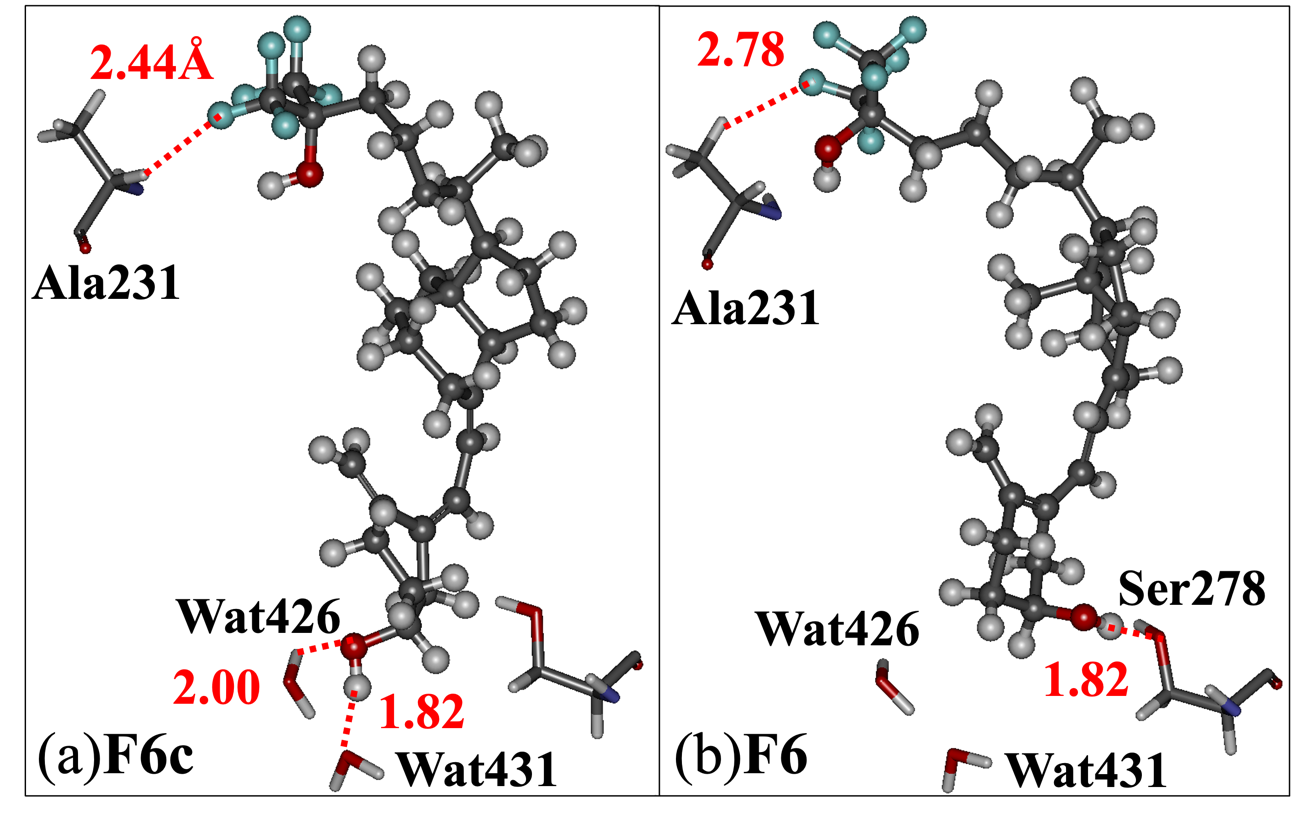


Figure S9 Interaction structures between critical VDR residues and compounds ((a) **F6c** and (b) **F6**) in the VDR−compound complexes. Red lines indicate the distances between atoms of the compound and VDR residue. The present FMO calculations indicate that F atoms of the terminal CF_3_ groups of **F6c** have +0.02 ~ +0.14 charges, while those of **F6** have +0.02 ~ +0.14 charges.


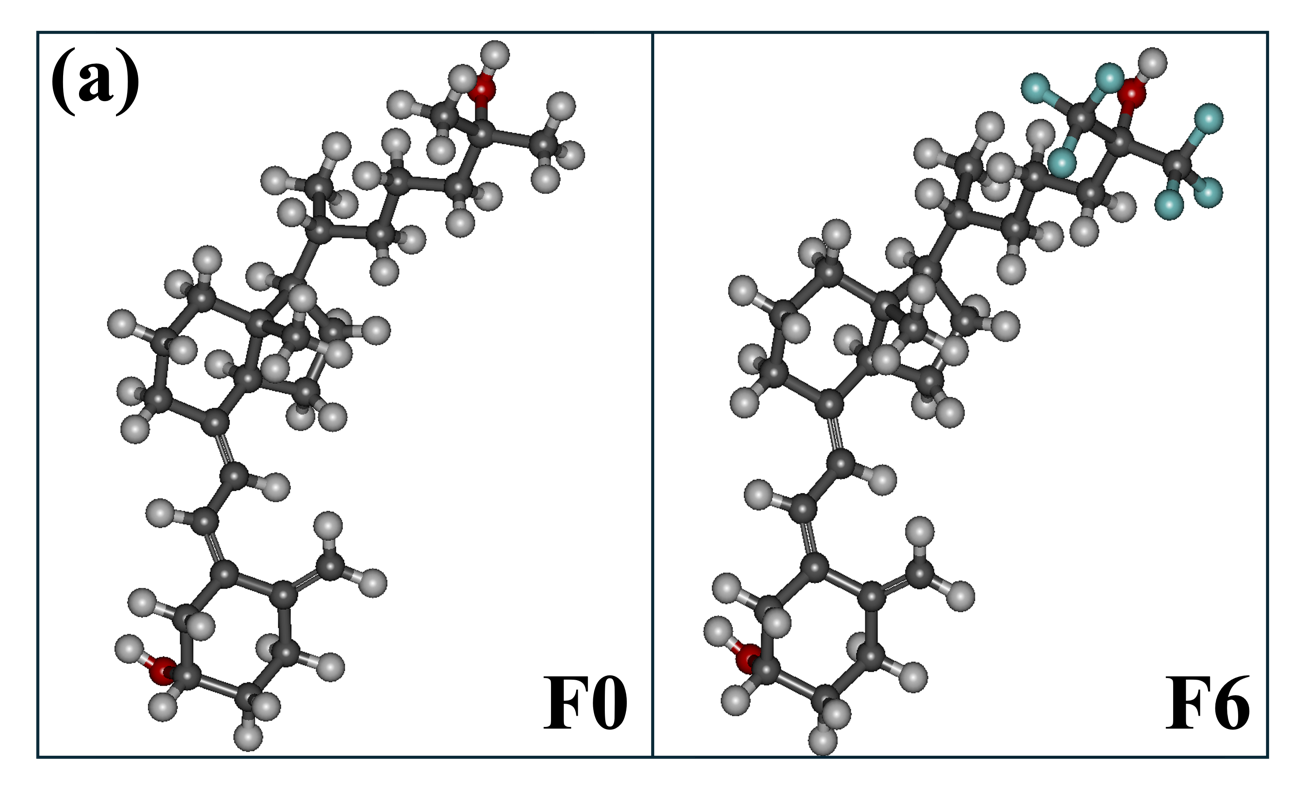


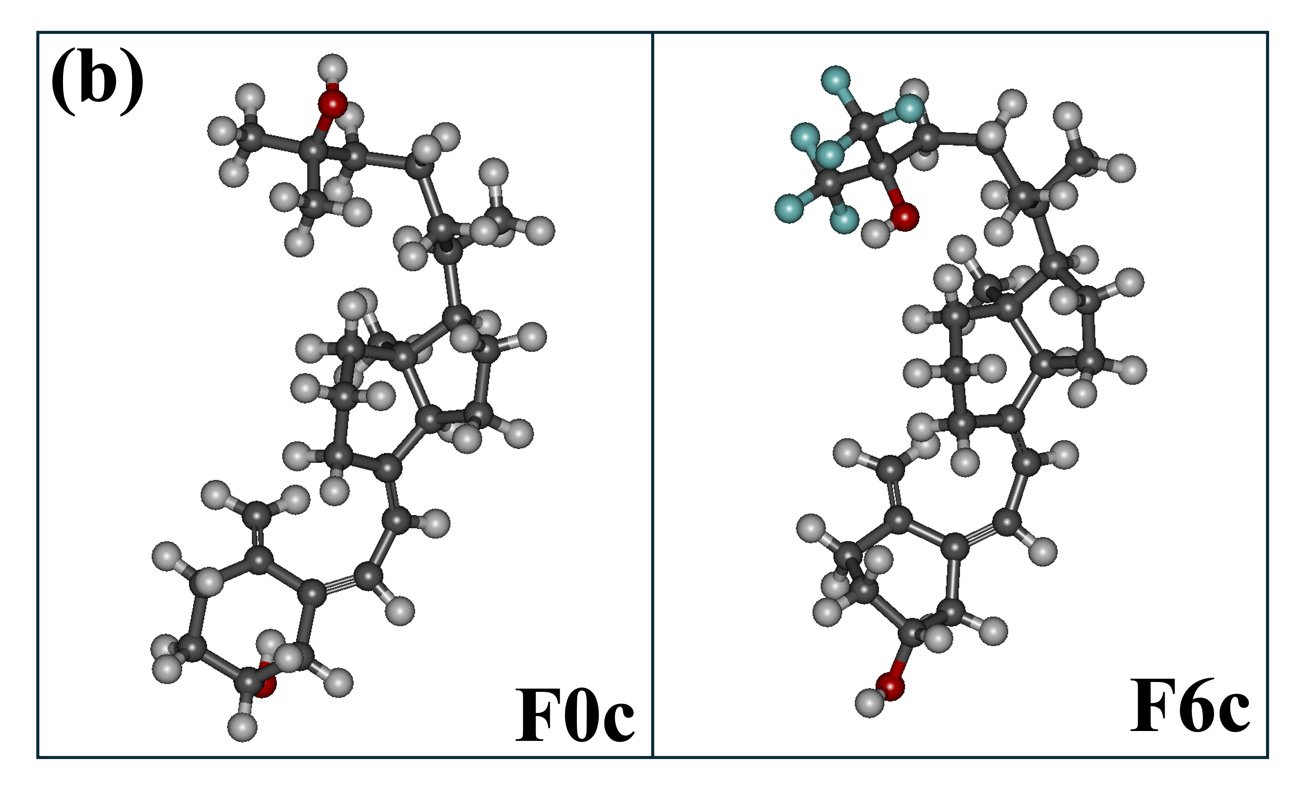


Figure S10 Optimized structures using MM method of (a) **F0** and **F6,** and (b) **F0c** and **F6c** in their complexes with VDR.
